# The complete mitochondrial genome of *Isochrysis galbana* harbors a unique repeat structure and a specific *trans*-spliced *cox1* gene

**DOI:** 10.3389/fmicb.2022.966219

**Published:** 2022-09-27

**Authors:** Jingping Fang, Xiuming Xu, Qinchang Chen, Aiting Lin, Shaoqing Lin, Wen Lei, Cairong Zhong, Yongji Huang, Yongjin He

**Affiliations:** ^1^College of Life Science, Fujian Normal University, Fuzhou, China; ^2^Key Laboratory of the Ministry of Education for Coastal and Wetland Ecosystems, College of the Environment and Ecology, Xiamen University, Xiamen, China; ^3^Center of Engineering Technology Research for Microalgae Germplasm Improvement of Fujian, Southern Institute of Oceanography, Fujian Normal University, Fuzhou, China; ^4^Fujian Provincial Key Laboratory of Haixia Applied Plant Systems Biology, Center for Genomics and Biotechnology, Fujian Agriculture and Forestry University, Fuzhou, China; ^5^College of Geography and Oceanography, Minjiang University, Fuzhou, China

**Keywords:** haptophytes, *Isochrysis galbana*, mitochondrial genome, comparative analysis, *trans*-splicing, RNA editing

## Abstract

The haptophyte *Isochrysis galbana* is considered as a promising source for food supplements due to its rich fucoxanthin and polyunsaturated fatty acids content. Here, the *I. galbana* mitochondrial genome (mitogenome) was sequenced using a combination of Illumina and PacBio sequencing platforms. This 39,258 bp circular mitogenome has a total of 46 genes, including 20 protein-coding genes, 24 tRNA genes and two rRNA genes. A large block of repeats (~12.7 kb) was segregated in one region of the mitogenome, accounting for almost one third of the total size. A *trans*-spliced gene *cox1* was first identified in *I. galbana* mitogenome and was verified by RNA-seq and DNA-seq data. The massive expansion of tandem repeat size and *cis*- to *trans*-splicing shift could be explained by the high mitogenome rearrangement rates in haptophytes. Strict SNP calling based on deep transcriptome sequencing data suggested the lack of RNA editing in both organelles in this species, consistent with previous studies in other algal lineages. To gain insight into haptophyte mitogenome evolution, a comparative analysis of mitogenomes within haptophytes and among eight main algal lineages was performed. A core gene set of 15 energy and metabolism genes is present in haptophyte mitogenomes, consisting of 1 *cob*, 3 *cox*, 7 *nad*, 2 *atp* and 2 ribosomal genes. Gene content and order was poorly conserved in this lineage. Haptophyte mitogenomes have lost many functional genes found in many other eukaryotes including *rps*/*rpl*, *sdh*, *tat*, *secY* genes, which make it contain the smallest gene set among all algal taxa. All these implied the rapid-evolving and more recently evolved mitogenomes of haptophytes compared to other algal lineages. The phylogenetic tree constructed by *cox1* genes of 204 algal mitogenomes yielded well-resolved internal relationships, providing new evidence for red-lineages that contained plastids of red algal secondary endosymbiotic origin. This newly assembled mitogenome will add to our knowledge of general trends in algal mitogenome evolution within haptophytes and among different algal taxa.

## Introduction

The haptophyte microalgae are typically single-celled phytoplankton with tiny cell size (2–20 μm) that live ubiquitously in the photic zone of the oceans and freshwater bodies. As main primary producers, the haptophytes alone may represent 30%–50% of marine photosynthetic biomass, playing a pivotal role in global CO_2_ fixation in a variety of aquatic ecosystems (Liu et al., 2009). Haptophytes and other three distinct and distantly related lineages including *c*ryptophytes, *a*lveolates, *s*tramenopiles (heterokonts) contained red algal secondary-derived plastids. These four groups were once collectively known as the chromalveolates or CASH based on a uniting biological feature of plastids, but their evolutionary history had long been a bone of contention (Cavalier-Smith, 1999; Petersen et al., 2014; Ponce-Toledo et al., 2019). Recently the monophyly of CASH was overturned by addition of other previously unrelated groups: *a*lveolates and *s*tramenopiles branches with *r*hizarians forming SAR supergroup (Keeling and Burki, 2019), even T-SAR clade with the subsequent addition of *t*elonemids (Strassert et al., 2019); *h*aptophytes have merged with centrohelids to form Haptista supergroup while *c*ryptophytes saw a coalescence of a few lineages (*Palplitomonas* and katablepharids) around them to form Cryptista (Keeling and Burki, 2019), even the newly proposed clade Pancrytista plus *Microheliella maris* (Yazaki et al., 2022). With the maturation of genomic methods and uncovering of new species, clarifying and confirming the phylogenetic positions of these major eukaryotic lineages will continue, which will facilitate the better understanding of distant and murky past of early evolution.

Mitochondria are known as the “powerhouse” of the eukaryotic cell as they are the site of aerobic respiration, generating energy-rich adenosine 5′-triphosphates (ATPs) that can be used to fuel the metabolic activities of organisms. About 1.4 billion years ago, mitochondria evolved through endosymbiosis, where free-living single-celled α-proteobacteria ancestors were engulfed by primitive cells and integrated into the host cell (Youle, 2019). Over the course of evolution, the endosymbiont “domestication” in the host cell involved a drastic reduction of genome size and coding capacity resulting from gene loss or massive early non-linear endosymbiont-to-nucleus gene migration events. This process of gene transfer to the nucleus occurred in waves of exponential reduction, in parallel and independently with lineage-specific rates, thus leading to multiple origins of mitochondria and varying degrees of gene retention (Janouškovec et al., 2017). The drastic elimination of redundant genes led to only 0.5%–1.2% of the initial gene repertories retained in present-day mitochondrial genomes (hereafter, mitogenomes; Burger et al., 2013). Mitogenomes in eukaryotes are in every shape and size, with large variations in size, genome architecture, gene order and content, the mobile genetic elements and repeat structure (Smith and Keeling, 2015; Nishimura et al., 2019). Compared with the small compact circular animal mitogenomes (36–37 genes), land-plant and algal mitogenomes possess a puzzling array of genome architectures: large in size and complex in non-coding regions with varying gene number. The most eubacteria-like and gene-rich eukaryotic mitogenomes reported to date are that of jakobids members, which harbor up to 100 genes (including 61–69 protein-coding genes; Burger et al., 2013). Conversely, apicomplexans and their relatives possess the smallest mitogenomes (~6 kb in size) with merely 3–5 genes (Flegontov et al., 2015).

In spite of the ecological and phylogenetic importance of haptophytes microalgae, advances in haptophytes genomics have lagged behind other major algal lineages due to the difficulty in excluding the contaminations of its symbiotic bacteria. As of 2016, around 312 haptophytes species had been morphologically characterized (Edvardsen et al., 2016), but the mitogenomes of only 13 haptophyte species have been sequenced, including *Emiliania huxleyi* (Puerta et al., 2004; Smith and Keeling, 2012) and four *Gephyrocapsa* species (Kao et al., 2022) from Isochrysidales, *Phaeocystis antarctica* (Smith et al., 2014), *Chrysochromulina tobin* (Hovde et al., 2014), *Chrysochromulina parva* (Hovde et al., 2019), *Chrysochromulina* sp. (Nishimura et al., 2014) and *Phaeocystis globosa* (Song et al., 2021) from Prymnesiales, *Pavlova lutheri* (Hulatt et al., 2020) and *Diacronema viridis* (Kim et al., 2021) from Pavlovales, and a novel alga *Pavlomulina ranunculiformis* NIES-3900 from a newly erected haptophyte class, Rappephyceae (Kawachi et al., 2021). The small mitogenome has been considered as an ideal model for genetic diversity, phylogenetic and comparative genomic analysis in algal species with improved resolution compared with traditional molecular markers (Kim et al., 2018; Sibbald et al., 2021; Starko et al., 2021; Zhang et al., 2021; Van Beveren et al., 2022). Unveiling more haptophyte mitogenomes would provide insight into the evolutionary history of haptophytes and the relationships among CASH lineages.

Extensive posttranscriptional modifications such as RNA editing and intron splicing are required for plant mitochondrial transcripts during converting RNA from nascent into mature state (Ichinose and Sugita, 2016; Guo et al., 2020). RNA editing is a process during transcription whereby specific nucleotides within mRNA and tRNA sequences were modified by insertions, deletions or base substitutions, thereby affecting the subsequent translation process (Steinhauser et al., 1999). RNA editing is an adaptive process to correct deleterious mutations in non-recombinant organellar genomes, and commonly observed in very diverse groups of eukaryotes, especially in higher plant mitochondria. *Trans*-splicing, whereby the two exons to be joined to form mature mRNA are from distinct transcripts, has mainly been found in plant organelles and prokaryotes (Bonen, 2008; Laroche-Johnston et al., 2018; Guo et al., 2020). Compared to the typical RNA *cis*-splicing event, which describes the joining of exons from the same primary transcript, *trans*-splicing is much less common. Outside of the green-algal lineage (Goldschmidt-Clermont et al., 1991; Cahoon et al., 2017; Kück and Schmitt, 2021), dinoflagellates (Jackson and Waller, 2013) and diplonemids (Valach et al., 2016), little is known about the RNA editing and *trans*-splicing events in haptophytes. The generation of haptophyte mitogenomes will also give us a good opportunity to undergo a thorough and comprehensive *in silico* survey of organelle transcriptomes of haptophytes to identify the RNA editing and *trans*-splicing events.

The unicellular golden-brown haptophyte *Isochrysis galbana* is a member of the Isochrysidaceae family of Isochrysidales order. It is considered as an ideal natural source for human and animal food supplements because it contains rich valuable bioactive compounds such as fucoxanthin (Zarekarizi et al., 2019) and polyunsaturated fatty acids (PUFA; Di Lena et al., 2020). Besides, the high saturated acid of *I. galbana* can contribute to the improved biodiesel quality applied in renewable energy systems (Sánchez et al., 2013; Silitonga et al., 2017). The small genome with low-level heterozygosity (~93 M), appropriate cell size (5–7 μm), cell wall-less feature, high growth rate and short generation time together make it an exceptionally promising microalgae model for genetic and genomic studies to address many biological questions. A high-quality nuclear genome and a complete chloroplast genome (cpDNA) of *I. galbana* have been reported (Fang et al., 2020; Chen et al., 2022). However, until recently little was known about the mitochondrial genomic characterization of *I. galbana*.

Mitogenomes are evolving much faster than their plastid genomes in three distinct lineages with secondary red plastids including haptophyte species (Smith and Keeling, 2012), thus often resulting in large complex repeat structures in algal mitogenomes. Nearly all published haptophyte mitogenomes contain complex and highly repetitive non-coding regions. For example, the mitogenome of haptophyte *C. tobin* has a 9 kb long repeat region, which features three ~1.5 kb large tandem repeats that are flanked by two regions containing small tandem repeats (Hovde et al., 2014). The first full-length mitogenome of haptophyte *P. globosa* strain CNS00066 contains two large repeat regions with combined length of 20.7 kb, representing the longest repeat region among sequenced haptophytes mitogenomes (Song et al., 2021). The accumulation of tandem repeats in large intergenic regions (LIRs) of mitogenomes are also ubiquitous in unicellular green algae (Turmel et al., 1999, 2007), red algae (Van Beveren et al., 2022), cryptophytes (Hauth et al., 2005; Kim et al., 2008) and diatoms (Oudot-Le Secq and Green, 2011). *Porphyridium* harbors the largest red algal mitogenomes reported thus far, which could be ascribed to the invasion of group II introns in genic regions and the repeat-rich LIRs (Kim et al., 2022). Undoubtedly, the complexity of repeat structures within mitogenomes in haptophytes and other related algae would present a challenge for assembling the complete circular mitogenomes. It can be inferred that incompletely assembled (linear) mitogenomes of several haptophyte species (*P. antarctica* [GenBank: JN131834, JN131835], *P. globosa* [GenBank: KC967226]*, Pavlova lutheri* [GenBank: HQ908424], *Gephyrocapsa species* [GenBank: OL703630- OL703635], *P. ranunculiformis* [GenBank: LC564891 plus LC564892]) could be attributed to the presence of one or more large complex repeat structures by which the short-read strategy is limited. Revolutionary breakthroughs in sequencing technologies and bioinformatics methods that are tailored to solve assembly difficulties have largely overcome the short-read dilemma. The Pacific BioSciences (PacBio) long reads combined with the correction of Illumina short-read data was proved to be a highly accurate method to assemble finished-quality (circularized) organellar genomes with no gaps (Fang et al., 2020; Song et al., 2021).

Here, the first complete *I. galbana* mitogenome was constructed based on a combination of PacBio RSII and Illumina Hiseq data from the ongoing *I. galbana* genome sequencing project. This complete circular mitogenome allows us to perform comparative mitogenome analysis of haptophytes and give a more holistic view of the gene content, architecture, arrangement and complex repeat structure among haptophyte species. We also used Illumina resequencing and transcriptome sequencing data to *in silico* screen the *trans*-splicing and RNA editing events in haptophytes for the first time. Our results of algal mitogenomes comparison and phylogenetic analysis provide us with a specific perspective of the evolutionary pattern of haptophytes and related algal lineages.

## Materials and methods

### Culture maintenance, genomic DNA isolation and sequencing

*Isochrysis galbana* OA3011 was deposited in the Southern Institute of Oceanography, Fujian Normal University, China. The *I. galbana* cultures were maintained in 250 ml Erlenmeyer flasks containing 100 ml f/2 medium10 and incubated at 23 ± 1°C under 100 μmol photons m^−2^ s^−1^ light on a 12 h:12 h light:dark cycle using fluorescent light bulbs. These flasks were shaken manually 4–6 times a day. Purified total genomic DNA was isolated using a modified cetyltrimethylammonium bromide (CTAB) method (Allen et al., 2006). The concentration and purity of DNA was evaluated by a NanoDrop 2000c spectrophotometer (Life Technologies, DE, United States). The *Isochrysis galbana* genome was sequenced using a combination of Illumina and PacBio sequencing technologies. Library construction and sequencing of *I. galbana* genome was carried out by the Novogene Company (Beijing, China). Resequencing was performed on Illumina HiSeq X Ten platform (Illumina Inc., CA, United States) in paired-end (PE) 150 nt mode. Prior to downstream analysis, raw Illumina data were initially subjected to quality checks to obtain clean reads. The empty reads, reads with low-quality bases [Phred quality score (Q) < 20] and Illumina adapters were filtered out by Trimmomatic v0.36 (Bolger et al., 2014). After filtering, over 8.92 Gbp clean PE data including 59.45 million high-quality reads were generated, which represented around 89× genome equivalents. For PacBio library construction and sequencing, at least 5 μl sheared and concentrated DNA was applied to size-selection with BluePippin (Sage Science, MA, United States). A total of ~15.5 Gb data composed of 2,033,745 million reads were obtained from the PacBio RSII platform, i.e., 166 × coverage of the estimated genome size.

### Mitochondrial genome assembly

The Illumina-generated reads were assembled by NOVOPlasty (Dierckxsens et al., 2016) with the mitogenome of closely related species *Emiliania huxleyi* (GenBank: AY342361.1) as reference genome, which produced a single linear contig of 27,129 bp as a candidate mitogenome. Based on homologous BLAST searches against the NOVOPlasty result and mitogenomes of related species *E. huxleyi* and *Chrysochromulina* sp. CCMP291 (GenBank: KJ201908.1), 310,630 potential mitochondrial reads consisting of 46,594,500 bp data were extracted from the Illumina reads pool. Those extracted Illumina homoreads were used to perform mitogenome *de novo* assembly using the Abyss (Simpson et al., 2009), SPAdes (Bankevich et al., 2012) and SOAPdenovo2 (Luo et al., 2015). The Abyss draft assembly that could cover the total length of NOVOPlasty result was chosen for further analysis. PacBio long-reads were aligned against the NOVOPlasty and Abyss assembled contigs using BLASR program (Chaisson and Tesler, 2012). Aligned PacBio reads were extracted from the reads pool and considered as potential chloroplast reads. A total of 6.52 Gb data composed of 869,453 million PacBio long reads was extracted after aligning, which were used to perform self-correction and mitogenome *de novo* assembly of cp genome using CANU v2.1 with default parameters (Koren et al., 2017), followed by error correction using the Quiver (Chin et al., 2013) and Pilon program (Walker et al., 2014). We check the circularity of the final assembly of mitogenome by the “check_circularity.pl” script provided by the sprai package.^1^ The resulting mitogenome assembly was arbitrarily reordered and oriented according to the mitogenome sequence of *E. huxleyi* which starts with genes *rrnL* and *rrnS* on the forward strand.

### Total RNA isolation, transcriptome sequencing and read mapping

When the *I. galbana* cells reached the logarithmic phase (10^6^ cells/ml), the culture was evenly divided into eight groups. Each two groups were cultured under white, green, blue and red light of 100 μmol photons m^−2^ s^−1^, respectively. The white light was used as control. These groups were harvested at 3 and 7 days. Three biological replicates were carried out for each treatment. Total RNA was extracted using Omega E.Z.N.A.^®^ Plant RNA Kit (Omega Bio-tek Inc., GA, United States) and purified using RNeasy MiniElute Cleanup Kit (Qiagen, Hilden, Germany) following the manufacturer’s instructions. After quantity and quality determination, RNA samples were further used to Illumina sequencing library construction. A total of 24 cDNA libraries (D3 and D7 samples under four different light qualities) were constructed and sequenced on the Illumina HiSeq X Ten platform (Illumina Inc., CA, United States) in paired-end (PE) 150 nt mode by the Novogene Company (Beijing, China). Prior to downstream analysis, raw Illumina data were initially subjected to quality checks to obtain clean reads. The empty reads, reads with low-quality bases [Phred quality score (Q) < 20] and Illumina adapters were filtered out by Trimmomatic v0.36 (Bolger et al., 2014). Quality reports for the raw and clean RNA-seq data are available in Supplementary Table S1.

Over 1.97 billion clean PE reads, totaling 295.59 Gb transcriptome data were generated. PE 150-bp reads were aligned with the *I. galbana* OA3011 mitochondrial, chloroplast and genomic sequences, respectively using HISAT2 v2.1.0 (Kim et al., 2015) with parameters: --new-summary -p -x -1 -2 -S. The reads for each biological replicate were mapped independently, during which Sequence Alignments/Map (SAM) format files were produced. Unmapped reads were removed in raw SAM files. Then two concatenated SAM files, one for mitochondria and the other for chloroplast, were converted to Binary Alignment/Map (BAM) format and sorted according to chromosomal coordinates using the SAMtools suite (Li et al., 2009). The DNA resequencing short and long reads generated from Illumina and PacBio platforms were aligned against the complete *I. galbana* mitogenome using BWA (Li and Durbin, 2009) and Minimap2 (Li, 2018), respectively. Multiple-mapped reads and PCR duplicates were removed to prevent the false positives. The Integrative Genomics Viewer (IGV) software (Thorvaldsdóttir et al., 2013) was used to manually visualize and check the accuracy of assembly, gene annotation and gene expression level using the BAM alignment output as a guide.

### Genome annotation and physical mapping

Preliminary annotation of protein-coding genes was based on *ab initio* gene predicitons by GeneMarkS (Besemer et al., 2001) and homologous predictions by BLAST searches (Altschul et al., 1990) against extracted gene sequences from published mitogenomes of five haptophyte species *Emiliania huxleyi* CCMP1516 (linear; GenBank: JN022704.1), *Emiliania huxleyi* (GenBank: AY342361.1), *Chrysochromulina parva* (GenBank: NC_036938.1), *Chrysochromulina* sp. NIES-1333 (GenBank: AB930144.1) and *Chrysochromulina tobin* CCMP291 (GenBank: KJ201908.1). The start/stop codons and intron/exon boundaries of each protein-coding gene were manually corrected in SnapGene Viewer^2^ by referencing the transcriptome alignment file and mitochondrial genes of related species. GroupII introns were predicted by the RNAweasel program (Lang et al., 2007). The noncoding RNA genes (ncRNAs) include transfer RNA genes (tRNAs) and ribosomal RNA genes (rRNAs). We predicted rRNAs by homologous gene evidence and transcript evidence, and tRNAs by tRNAscan-SE version 2.0.4 (Schattner et al., 2005) with default parameters. Manual inspection was also performed to remove overlapped ncRNAs and remain the longest ones with high-confidence. Tandem Repeats Finder (v4.10; Benson, 1999) was applied to identify tandem repeats. The circle graph of *I. galbana* mitogenome was drawn by Circos v0.69–9 (Krzywinski et al., 2009).

Functional annotation of the protein-coding genes was carried out by BLASTP based on sequence-similarity searches against five publicly available protein databases: NCBI non-redundant protein database (Nr), Gene Ontology (GO), Kyoto Encyclopedia of Genes and Genomes (KEGG), Clusters of Orthologous Groups (COGs) and Swiss-Prot, with a typical *E*-value cut-off of 1e−5.

### Codon usage and RNA editing detection

Codon usage and relative synonymous codon usage (RSCU) were analyzed by CUSP program in EMBOSS.^3^ For the analysis of RNA editing, the “mpileup” utility of SAMtools software suite (Li et al., 2009) was performed to call SNP variants with parameters -I -C50 -u -q 20 -Q 20 based on the transcript alignment output (the BAM file) aforementioned, followed by of SAMtools “bcftools” command. A minimum variant frequency threshold of 0.1 was set to minimize the possibility of overlooking edits due to a low editing frequency. The output variant call format (VCF) file contained all polymorphism information between mRNA transcripts and the DNA sequence. The raw variant calls were filtered with the SAMtools vcfutils.pl. varFilter script and a python script vcf_filter.py for read depth ≥ 5 and polymorphism site quality ≥50. The final SNPs and InDels in the filtered VCF file represent the putative RNA editing sites. The editing efficiency of each site was estimated by calculating the proportion of RNA resequencing reads that contained the SNPs.

### Comparative mitochondrial genomic analysis

The complete sequences and genbank files of 10 haptophyte mitochondrial genomes were downloaded from NCBI Genbank, including *Emiliania huxleyi* CCMP1516 (linear; GenBank: JN022704.1), *Emiliania huxleyi* CCMP373 (GenBank: AY342361.1), *Diacronema viridis* voucher KMMCC0113 (GenBank: MW044630.1), *Diacronema viridis* culture CCMP620 (GenBank: MW044629.1), *Pavlova* sp. NIVA-4/92 (GenBank: MN564259.1), *Phaeocystis globosa* CNS00066 (GenBank: MW435860.1), *Phaeocystis antarctica* CCMP1374 (GenBank: JN131834.1), *Chrysochromulina parva* (GenBank: NC_036938.1), *Chrysochromulina* sp. NIES-1333 (GenBank: AB930144.1) and *Chrysochromulina tobin* CCMP291 (GenBank: KJ201908.1). Multiple sequence alignment of mitochondrial genomes of *I. galbana* with other 10 haptophyte algae was conducted on AliTV software (Ankenbrand et al., 2017). The OrthoMCL program (Li et al., 2003) was used to identify common single-copy orthologous genes in 11 mitogenomes with an *E*-value cutoff of 1e-5. The maximum-likelihood (ML) phylogenetic tree was inferred by PhyML v3.0 (Guindon et al., 2010) employing 1,000 bootstrap replicates and the LG + I + G + F model for amino acid sequences. Mauve genome aligner (Darling et al., 2004) was used to assess the extent of mitochondrial genome rearrangements of haptophyte mitogenomes.

Comparisons among all known mitogenomes from a wide range of algal lineages have been made. We collected a total of 2,942 mitogenomes that have been published to date (08/08/2021) of nine main lineages in NCBI, including 333, 282 and 12 mitogenomes from three primary algal lineages (green algae, red algae, glaucophytes), 22, 1,732, 529, 38 mitogenomes from four chlorophyll-*c* containing algal lineages (*c*ryptophytes, *a*lveolates, *s*tramenopiles, *h*aptophytes), one mitogenome (*Lotharella oceanica*) from green alga-derived lineage (chlorarachniophytes) and 20 mitogenomes from the jakobids group. Euglenophytes and cyanophytes were not included in this analysis due to no available data of mitogenomes in Genbank. The core set of genes from each lineage were inferred based on gff3 annotation files of all mitogenomes published to date. An in-house python script was used to dig out the conserved core gene set in each group based on the concatenated gff3 file.

### Divergence of coding gene sequences

To identify positive and negative selection in Isochrysidales species, nonsynonymous (*K*a) and synonymous (*K*s) substitution rates of 19 functional protein-coding genes shared by three species (*I. galbana* OA3011, *E. huxleyi* CCMP373 and *E. huxleyi* CCMP1516) were calculated. Novel genes (ORFs) were excluded from this analysis. Sequences of these 19 shared exons were extracted from three mitogenomes using an in-house Python script. Each exon of *E. huxleyi* was aligned separately with the same exon of *I. galbana* as reference using ClustalW2 (Larkin et al., 2007). To evaluate the divergence of paralogous genes, the KaKs_Calculator tool (Zhang et al., 2006) with parameters “-c 11 -m MS” was performed to calculate the *K*a, *K*s and evolutionary constraint (*K*a/*K*s rate) between paralogous pairs of genes based on the output alignment file from ClustalW2. *Ka*/*Ks* value of >1 signifies the gene is subjected to positive selection; *Ka*/*Ks* value of 1 indicates neutral selection; *Ka*/*Ks* value of <1 represents negative purifying selection.

### Phylogenetic analysis

The *cox1* gene as a single copy gene is conserved and present in the great mass of algal mitogenomes, and thus was used to investigate the evolutionary pattern of mitochondrial genes among seven algal lineages. The mitogenomes of 200 species (204 mitogenomes in total) in green-algal lineage Chlorophyta (25 species) and Cercozoa (1 species), Glaucophyta (6 species), and five red-algal lineages consisting of Cryptophyta (10 species), Alveolata (13 species), Stramenopiles (79 species) and Haptophyta (15 species) and Rhodophyta (51 species) were retrieved from NCBI GenBank. The mitogenomes from Cryptophyta, Haptophyta, Cercozoa and Glaucophyta nearly covered all available species in GenBank and most mitogenomes in other phyla we selected were recently published before the June, 2022. All selected mitogenomes were checked to ensure that the *cox1* gene was a single-copy gene. The coding sequences of *cox1* genes were extracted by an in-house perl script “getSeqFromList.pl” and transferred the coding sequences to amino acid sequnences then aligned using MUSCLE v.3.8.1 with default parameters (Edgar, 2004). Multiple sequence alignments were manually trimmed to exclude ambiguously aligned areas adjacent to indels. The ML phylogenetic tree of mitochondrial *cox1* genes was inferred using IQ-TREE 2 v2.1.4-beta (Minh et al., 2020). The best-fitting substitution model of ML for *cox1* was assessed to be “TN + F + R10” according to the Bayesian information criterion (BIC) by “-m MFP” parameter. Branch supports were calculated using 1,000 ultrafast bootstrap replicates and 1,000 replicates of SH-aLRT test (“-alrt” parameter; Guindon et al., 2010).

The concatenated mitochondrial dataset comprised 10 common single-protein (nad1, nad2, nad3, nad4, nad4L, nad5, nad6, cob, cox1, atp6) among 178 species (183 mitogenomes) and 6,266 amino acid positions in total. The mitogenomes of 178 species in green-algal lineage Chlorophyta (24 species) and Cercozoa (1 species), Glaucophyta (6 species), and four red-algal lineages consisting of Cryptophyta (7 species), Stramenopiles (77 species) and Haptophyta (13 species) and Rhodophyta (50 species) were retrieved from NCBI GenBank. Alignments were trimmed by Muscle v.3.8.1 program with default parameters and merged into a single matrix. The ML phylogeny was computed in IQ-TREE 2 v2.1.4-beta. The best-fit model “mtInv + F + I + I + R10” was generated by “-m MFP” and branch supports were calculated using 1,000 ultrafast bootstrap replicates and 1,000 replicates of SH-aLRT test.

## Results and discussion

### *Isochrysis galbana* mitogenome gene content, annotation and codon usage

**Figure 1:**
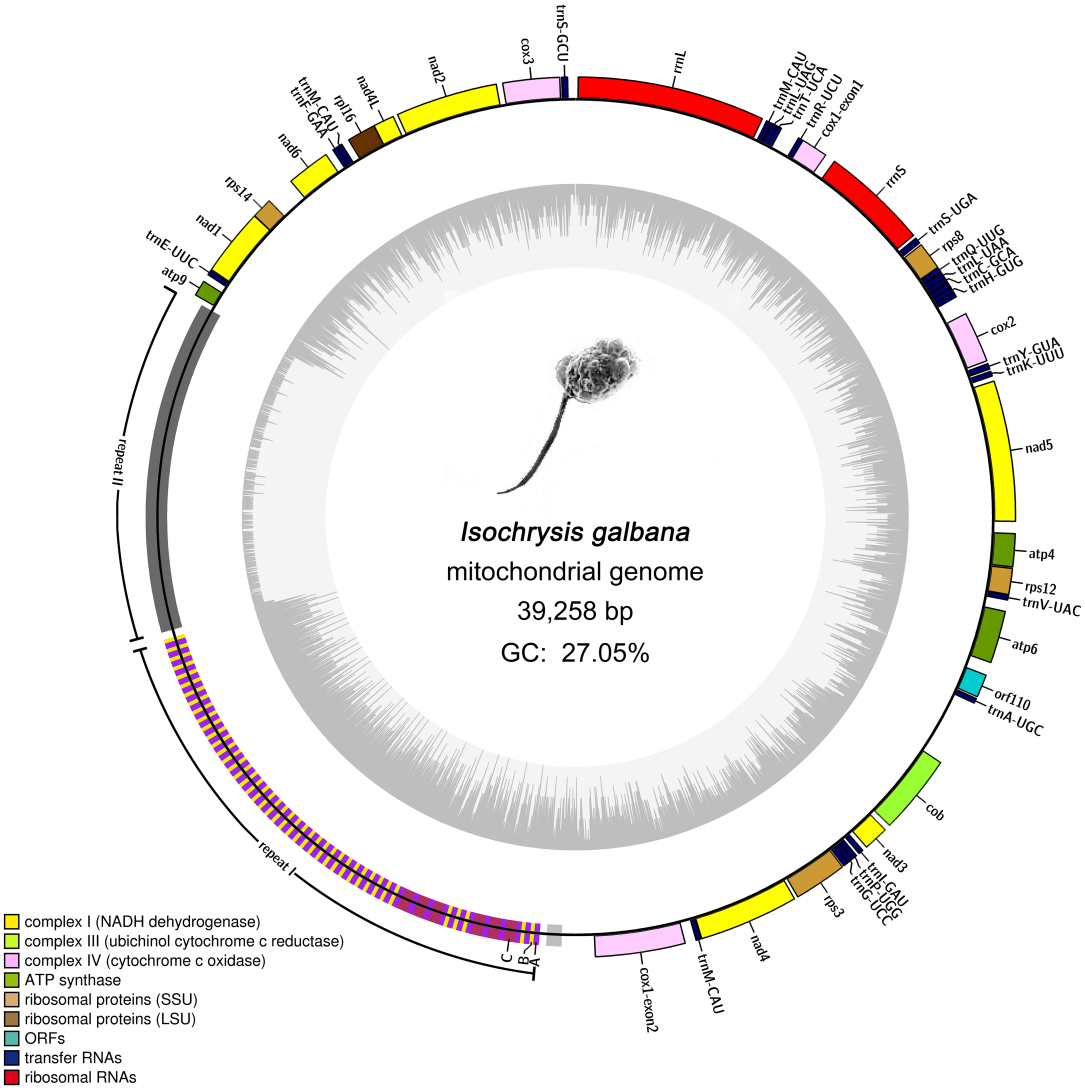
Mitochondrial genome map of *Isochrysis galbana* (Isochrysidaceae). Genes facing outside of the circle are transcribed in the clockwise directions. All genes are transcribed in the same direction. Genes are colored according to different functional groups. A split ribosomal operon is present, comprising genes encoding small (*rrnS*) and large (*rrnL*) subunits of rRNAs; No 5S rRNA gene was detected. The large block of repeat region (12.7 kb) represents a significant portion (~32.24%) of the entire genome. Detailed representation of the large complex repeat region is shown. The whole repeat region could be arbitrarily divided into two large tandem repeats (repeat I and repeat II) and one small flanked tandem repeat. Repeat I was composed of three subunits designated A, B, and C. Blocks A, B, and C have rarely perfect sequence identity.

The final complete mitogenome of *I. galbana* was assembled into a single circular double-stranded DNA molecule of 39,258 bp in length with an overall AT content of 72.95% (Figure 1). The mitogenome with gene annotation has been deposited in the NCBI GenBank database with the accession number ON688523. This mitogenome contained a large block of repeat sequences (~12.7 kb) segregated in one region of the genome, which accounted for almost one third of the total genome size (39.26 kb). Aside from the repeat region, this genome presented relatively compact in the coding region. The mitogenome encoded 46 genes, 44 of which were unique, including 20 for protein coding genes, 24 for tRNAs (22 unique) and a split ribosomal operon comprising genes encoding small (16S or *rrnS*) and large (23S or *rrnL*) subunits of rRNAs; no 5S rRNA gene was detected (Figure 1). One tRNA gene (*trnM-CAU*) was tripled and scattered singly in the mitogenome. All annotated genes were found to be encoded on a single strand. The 20 protein coding genes include seven, one, three and three (14 genes) encoding mitochondrial respiratory chain complexes I, III, IV, and V, respectively; one and four encoding large and small subunit ribosomal proteins, respectively. It is noteworthy that no genes were found to be related to complex II (the succinate dehydrogenase, secY) and cytochrome *c* biogenesis. A single novel gene with unknown function named *orf110* was identified. The open reading frame (ORF) of *orf110* contained 333 nucleotides, encoding a putative protein of 110 amino acids that had 66% similarity to the *orf104* gene of *Emiliania huxleyi* (GenBank: AAP94716.1) in Nr database. All the protein-coding genes had typical ATG start codon, except for the *orf110*, which contained the unusual TTA as an initiator codon. Coding regions with combined length of 21,643 bp comprised protein-coding genes (15,594 bp), tRNA genes (1,803 bp) and rRNA genes (4,246 bp), accounting for 55.14% of the genome, whereas the non-coding regions represented 44.86% of the genome. More specifically, the lengths of the *I. galbana* mitochondrial protein-coding genes ranged from 225 to 2,025 bp with an average length of 780 bp. The lengths of tRNA genes had an average length of 75 bp, ranging from 69 to 90 bp. The two rRNA genes (*rrnS* and *rrnL*) were, respectively, 1,544 and 2,702 bp in length. Detailed information on the *I. galbana* mitochondrial genes is provided in Supplementary Table S2.

Global functional analyses of all 20 protein-coding genes revealed that a total of 20, 12, 15, 8, and 15 genes were annotated to Nr, GO, COG, KEGG and Swiss-Prot databases, respectively (Supplementary Figure S1; Supplementary Tables S3–S5). Five genes were annotated in all databases and 12 corresponding genes were assigned with at least one GO term, which could be classified into three main ontologies: molecular function (MF), biological process (BP) and cellular component (CC; Supplementary Figure S1; Supplementary Table S4). Eight genes were mainly involved in metabolic, oxidative phosphorylation and ribosome pathways in our KEGG analysis (Supplementary Figure S2; Supplementary Table S5), which inosculates with the known mitochondrial function.

The potential codon bias and codon-anticodon recognition pattern in the *I. galbana* mitogenome was accessed (Supplementary Table S6). In total, 5,205 codons encoding 20 protein-coding genes were identified in the mitogenome. The 24 tRNAs consisting of 22 unique tRNA contained codons for 20 essential amino acid for algae biosynthesis. The most ubiquitous amino acid was leucine (758, 14.56%), followed by phenylalanine (514, 9.87%) and isoleucine (493, 9.47%), whereas tryptophane (6, 0.12%) was the least common amino acid. Relative synonymous codon usage (RSCU) was calculated by the coding sequences of 20 protein-coding genes in *I. galbana*. Based on the A/T position in codons, we found that A/T content located at the third position of each codon was the most common pattern (83.13%), followed by the second position of 65.80% and the first position of 64.44%. The RSCU analysis also present the similar trend that A/T were more frequently used (>1) compared with G/C at the third positions of *I. galbana* mitochondrial codons (Supplementary Table S6). This significant AT-rich bias at the third codon position is common in the extremely AT-rich biased organelle genomes.

### The large block of repeat region

Almost all sequenced haptophyte mitogenomes contain highly repetitive non-coding repeat regions, the exception being one of the Prymnesiale species *Chrysochromulina parva* (Table 1). Like other haptophytes, the complex and highly repetitive non-coding region contribute to the large genome size of *I. galbana* mitochondrion. The *I. galbana* mitogenome contained a large repeat localized to a single region measuring 12.7 kb in length (Figure 1), covering 32.24% of its genome. The Tandem Repeat Finder program (Benson, 1999) identified nine tandem repeat blocks which could be condensed to five blocks due to the presence of overlapping (Supplementary Table S7). These five blocks were comprised of repeat units ranging in size from 17 to 1,141 bp and were present in 2 to 115 copies. After manually checking, the whole repeat region could be arbitrarily divided into two large tandem repeats and one small tandem repeat (Figure 1), ~7.5 kb in length of the first large repeat (repeat I: 20,165–27,632) which was combined by several tandem repeat blocks and ~5.0 kb in length of the second large repeat (repeat II: 27,743–32,669) which was previously identified by Tandem Repeat Finder. The first large tandem repeat region (repeat I) was composed of three subunit classes, arbitrarily classified A, B and C, based on the sequence similarity within each subunit with high cutoff criteria of 95% identity (Figure 1). An additional small tandem repeat measuring ~0.3 kb whose unit had some homology to A and C subunits was located upstream of repeat I. Repeat unit A, unit B and unit C was comprised of 81, 56, and 159 bp in length with 50, 44, and 6 copies, respectively (Supplementary Table S8). Repeat I was primarily formed with two consistent patterns of A–B and A–C. The A–B pattern was the main pattern with a total length of 6,514 bp which accounted for 87.23% of repeat I, while A–C pattern represented 12.77% of repeat I with a total length of 954 bp.

**Table 1:** Comparison and characteristics of haptophyte mitochondrial genomes.

	*Isochrysis galbana*	*Emiliania huxleyi* CCMP1516 (linear)	*Emiliania huxleyi*	*Chrysochromulina parva*	*Chrysochromulina* sp. NIES-1333	*Chrysochromulina tobin* CCMP291	*Phaeocystis globosa* CNS00066	*Diacronema viridis* voucher KMMCC0113	*Diacronema viridis* culture CCMP620	*Pavlova* sp. NIVA-4/92
Order	Isochrysidales	Isochrysidales	Isochrysidales	Prymnesiales	Prymnesiales	Prymnesiales	Phaeocystales	Pavlovales	Pavlovales	Pavlovales
GenBank accession	This article	JN022704.1	AY342361.1	NC_036938.1	AB930144.1	KJ201908.1	MW435860.1	MW044630.1	MW044629.1	MN564259.1
Genome Size (bp)	39,258	28,660	29,013	24,009	34,291	34,288	43,585	29,282	29,282	36,202
GC%	27.09	28.5	28.31	32.47	29.96	31.36	29.35	39.15	39.18	37.46
Total genes (include RNAs)	46	48	48	48	47	49	46	47	47	49
Gene direction (+/−)	46/0	48/0	48/0	48/0	47/0	49/0	41/5	31/16	31/16	32/17
Protein-coding genes										
No. of protein-coding genes	20	20	21	20	17	21	19	20	20	22
Respiratory coding proteins	14	15	15	15	13	15	15	15	15	15
Ribosomal proteins	5	5	5	5	2	5	4	5	5	5
Core genes in all taxa	*atp6, atp9, cob, cox1, cox2, cox3, nad1, nad2, nad3, nad4, nad4L, nad5, nad6, rpl16, rps12*									
Unique gene content	2 (*orf110*, *trnT-UCA*)	1 (*trnI-CAU*)	1 (*orf104*)	0	3 (*orf584, orf627, trnS-ACU*)	1 (*orf457*)	0	0	0	2 (*orf 105*, *orf 636*)
Missing genes found in other haptophytes	4 (*atp8, dam, rpl14, rps19*)	3 (*atp8, rpl14, rps19*)	3 (*atp8, rpl14, rps19*)	3 (*dam, rpl14, rps19*)	8 (*atp4, atp8, dam, rpl14, rps14, rps19, rps3, rps8*)	3 (*dam, rpl14, rps19*)	4 (*dam, rpl14, rps19, rps8*)	3 (*dam, rps3, rps8*)	3 (*dam, rps3, rps8*)	3 (*dam, rps3, rps8*)
Unknow ORFs	1 (*orf110*)	0	1 (*orf104*)	0	2 (*orf584, orf627*)	1 (*orf457*)	0	0	0	2 (*orf 105, orf 636*)
RNA-coding genes										
tRNAs	24	25	25	26	28	26	25	24	24	24
rRNAs	2	2	2	2	2	2	2	3	3	3
rRNA content	1 split operon	1 intact operon	1 intact operon	1 split operon	1 split operon	1 split operon	1 split operon	1 split operon	1 split operon	1 split operon
Repeat elements										
Repeat blocks	9	4	5	0	5	7	15	8	8	19
Repeat size (kb)	12.66	1.88	2.29	0	0.24	1.88	19.62	1.69	2.21	3.67

No intron was found within listed mt genomes except for *Isochrysis galbana*.

The GC content of repeat I was 38.43%, which was much higher than that of the whole genome (27.05%), while only 6.9% was detected in the repeat II area. The increase size of tandem repeats in *I. galbana* and *P. globose* result in mitogenomes that are only 55% coding regions, in contrast to Jakobids species whose mitogenomes contained very high proportion of coding regions (80%–93%; Burger et al., 2013). The red algae *Chondrus crispus* owns the most compact mitogenome so far known, with coding sequences amounting to nearly 96%. The most loosely compact mitogenome and massive expansion in tandem repeats have also been reported in green alga *Chlorokybus atmophyticus* (Turmel et al., 2007) and the red alga *Porphyridium purpureum* (~132 kbp; Kim et al., 2022). As found in cryptophytes and most other algae (Kim et al., 2018), the tandem repeats within haptophyte mitogenomes represent species-specific pattern. The repeat sequences within the mitogenome of *I. galbana* showed no sequence and structural similarity to any tandem repeat block identified in other haptophyte mitogenomes. The origins of these repeat sequences still remain enigmatic. This is in contrast to mitogenomes in higher plants and animals whose tandem repeats play an important role in uncovering their evolutionary origins among species (Casane et al., 1997). It is increasingly thought that these variable repeats occurred as a result of strand slippage during recombination (Sibbald et al., 2021). The change in various repeat regions may be mainly induced by differential repeat unit amplification, which represents an indispensable driving force for algal mitogenome evolution (Song et al., 2021).

### Mitochondrial gene arrangement among haptophytes

We compared nine complete and circular mitogenomes of haptophycean algae sequenced to date which belong to four orders: Isochrysidales, Pavlovales, Phaeocystales and Prymnesiales (Table 1). The haptophyte mitogenomes ranged in length from 24,009 bp (*Chrysochromulina parva*) to 43,585 bp (*Phaeocystis globosa*). The size increase of the *P. globosa* mitogenome relative to the other haptophytes was largely attributed to an increase in repetitive elements, which reached up to nearly 19.62 kb. The mitogenome of *Chrysochromulina* sp. NIES-1333 in Prymnesiales owned the fewest number of protein-coding genes (17 genes), whereas *Pavlova* sp. in Pavlovales had the most (22 genes). Other species had 19–21 mt protein-coding genes. Comparison of the gene content among 37 available haptophyte mitogenomes revealed that a total of 24 protein-coding genes with known functions in addition to 8 novel genes were found (Supplementary Table S9). All haptophyte genomes contained an identical complement of 15 energy and metabolism genes consisting of one Cytochrome *b* gene (*cob*), 3 Cytochrome *c* oxidase genes (*cox1*, *cox2*, *cox3*), 7 NADH dehydrogenase genes (*nad1*, *nad2*, *nad3*, *nad4*, *nad4L*, *nad5*, *nad6*), 2 ATPase genes (*atp6*, *atp9*), and 2 ribosomal genes (*rpl16*, *rps12*; Table 1). Three genes *atp8*, *rpl14* and *rps19* were missing from all Isochrysidales mitogenomes, of which *rpl14* and *rps19* were found to be present exclusively in all Pavlovales species. The *nad9* gene was only present in *P. ranunculiformis* in the new class Rappephyceae. *Dam* gene which is responsible for DNA adenine methylation was only found in *E. huxleyi* and *G. oceanica* in all haptophyte mitogenomes (Supplementary Table S9).

With regard to rRNAs, most haptophytean mitogenomes contained only two rRNA genes (16S and 23S) except for Pavlovales which also had a 5S rRNA gene. The number of tRNAs ranged from 24 to 28 with some small variations in tRNA gene content (Table 2). For instance, *trnT-UCA* was only present in the order Isochrysidales and Prymnesiales. *trnU-UCA* was only found in Prymnesiales and Pavlovales, whereas *trnW-UCA* was exclusively present in the other two orders Isochrysidales and Phaeocystales. The Pavlovales had a unique isotype *trnW-CCA* while the *trnC-GCA* and *trnV-UAC* were absent in this order. *trnI-CAU* was missing in all listed species with the exception of *Emiliania huxleyi* CCMP1516. Likewise, *trnS-ACU* was only present in *Chrysochromulina* sp. NIES-1333. At least three copies of *trnM-CAU* were present in all examined species, suggesting a major role of this tRNA in haptophyte mitogenomes. It should be noted that *trnN-GUU* contained the codon for asparagine was absent in *I. galbana* although it could be found in any other known haptophyte mitogenomes.

**Table 2:** Mitochondrial transfer RNAs (tRNAs) and rRNAs in Haptophyta.

	*Isochrysis galbana*	*Emiliania huxleyi CCMP1516 (linear)*	*Emiliania huxleyi*	*Chrysochromulina parva*	*Chrysochromulina sp. NIES-1333*	*Chrysochromulina tobin CCMP291*	*Phaeocystis globosa CNS00066*	*Diacronema viridis voucher KMMCC 0113*	*Diacronema viridis culture CCMP620*	*Pavlova sp. NIVA-4/92*
**Order**	Isochrysidales	Isochrysidales	Isochrysidales	Prymnesiales	Prymnesiales	Prymnesiales	Phaeocystales	Pavlovales	Pavlovales	Pavlovales
*rrn5*	0	0	0	0	0	0	0	1	1	1
*rrnS*	1	1	1	1	1	1	1	1	1	1
*rrnL*	1	1	1	1	1	1	1	1	1	1
*trnA-UGC*	1	1	1	1	0	1	1	1	1	1
*trnC-GCA*	1	1	1	1	0	1	1	0	0	0
*trnD-GUC*	1	1	1	1	1	1	1	1	1	1
*trnE-UUC*	1	1	1	1	1	1	1	1	1	1
*trnF-GAA*	1	1	1	1	1	1	1	1	1	1
*trnG-UCC*	1	1	1	1	1	1	1	1	1	1
*trnH-GUG*	1	1	1	1	1	1	1	1	1	1
*trnI-CAU*	0	**1**	0	0	0	0	0	0	0	0
*trnI-GAU*	1	1	1	1	1	1	1	1	1	1
*trnK-UUU*	1	1	1	1	1	1	1	1	1	1
*trnL-UAA*	1	1	1	1	1	1	1	1	1	1
*trnL-UAG*	1	1	1	1	1	1	1	1	1	1
*trnM-CAU*	**3**	**2**	**3**	**3**	**6**	**3**	**3**	**3**	**3**	**3**
*trnN-GUU*	0	1	1	1	1	1	1	1	1	1
*trnP-UGG*	1	1	1	1	1	1	1	1	1	1
*trnQ-UUG*	1	1	1	1	1	1	1	1	1	1
*trnR-ACG*	1	1	1	1	1	1	1	1	1	1
*trnR-UCU*	1	1	1	1	1	1	1	1	1	1
*trnS-ACU*	0	0	0	0	**1**	0	0	0	0	0
*trnS-GCU*	1	1	1	1	1	1	1	1	1	1
*trnS-UGA*	1	1	1	1	1	1	1	1	1	1
*trnT-UCA*	1	0	0	1	1	1	0	0	0	0
*trnT-UGU*	1	1	1	1	1	1	1	1	1	1
*trnU-UCA*	0	0	0	1	1	1	0	1	1	1
*trnV-UAC*	1	1	1	1	1	1	1	0	0	0
*trnW-CCA*	0	0	0	0	0	0	0	**1**	**1**	**1**
*trnW-UCA*	0	1	1	0	0	0	1	0	0	0
*trnY-GUA*	1	1	1	1	1	1	1	1	1	1
**Total tRNAs**	24	25	25	26	28	26	25	24	24	24

In terms of gene strand directions, the haptophyte mitogenomes present variation of strand polarity within orders (Table 1), which are structurally similar to that of cryptophytes (Kim et al., 2018). All species in Isochrysidales and Prymnesiales showed absolute strand polarity but not in Pavlovales and Phaeocystales. To be more specific, all genes were located on the same strand in the *Isochrysis galbana*, *Emiliania huxleyi* and three *Chrysochromulina* sp. mitogenomes, while some genes were located on the opposite strand in *Diacronema viridis* (16 genes), *Pavlova* sp. (17 genes) and *Phaeocystis globose* (5 genes).

**Figure 2:**
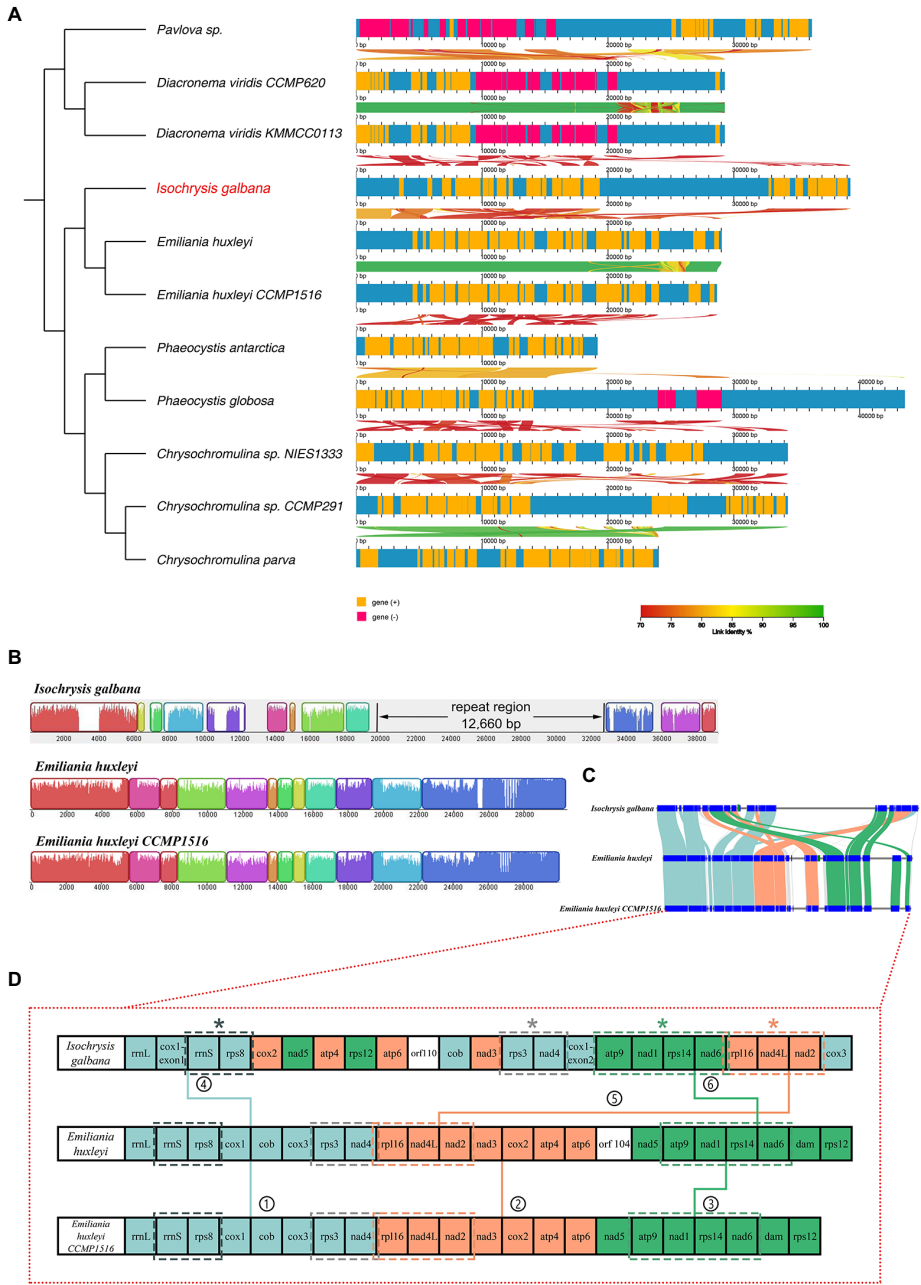
Syntenic comparisons of haptophyte mitochondrial genomes. **(A)** Syntenic comparisons of linear mitochondrial maps relative to a phylogenetic tree of 11 haptophyte species using AliTV software. Both panels depict pairwise comparisons, expressed as percentage of nucleotide similarity, that connect different homologous genomic regions. The x-axis denotes the site of the feature on the mitochondrial genome. An ML phylogenomic tree constructed with shared single-copy genes from 11 mitogenomes is shown on the left; **(B)** Gene map comparison of *I. galbana* and two *E. huxleyi* species in Isochrysidales order aligned using Mauve. A sequence identity similarity profile is shown inside each block. Three mitochondrial genomes were linearized starting at the large ribosomal operon *rrnL* gene. The ~12.6 kb tandem repeat region unique to *I. galbana* mitochondrial genome is shown in its linear map. **(C)** Synteny comparison of the *I. galbana* and two *E. huxleyi* species in Isochrysidales order. Light blue, orange and green color blocks represented three large conserved syntenic clusters of protein-coding genes, each cluster containing identical gene order among three species. **(D)** Gene content and arrangement of three Isochrysidales species. Three large synthetic blocks of protein-coding genes are represented as light blue, orange and green colors, respectively and indicated with numbers (1–6). Four conserved tandem gene clusters consisting of 2–4 genes among three species marked with asterisks.

With respect to co-linearity in gene placement among 11 haptophyte mitogenomes, many structural rearrangements have taken placed (Figure 2). In this section, the linear mitogenomes of *Emiliania huxleyi* CCMP 1516 and *Phaeocystis antarctica* CCMP1374 were also included in spite that they were partially assembled. These haptophyte species herein could be divided into four orders based on their mitogenome sequences, consisting of Isochrysidales, Prymnesiales, Phaeocystales and Pavlovales (Table 1; Figure 2A; Supplementary Figure S3), which is in accordance with traditional taxonomy. The most significant feature of the haptophyte mitogenomes was that their gene content and gene order were highly variable (Supplementary Table S9; Figure 2A; Supplementary Figure S3). The gene map comparison result showed that barely no syntenic gene blocks were arranged together among all haptophyte mitogenomes. Gene content was broadly conserved but gene order shuffled within the order of Isochrysidales which contained *I. galbana* and *E. huxleyi* (Table 1; Figures 2B–D), reflecting their close phylogenetic relationship. Mauve alignment between two *E. huxleyi* mitogenomes reflected they shared near perfect synteny with identical gene arrangements along the entire length other than the unique novel gene *orf104*. In contrast, *I. galbana* shared 11 locally syntenic blocks with the other two *E. huxleyi* mitogenomes (Figure 2B), which could arbitrarily be assigned into three large conserved syntenic clusters of protein-coding genes (Figures 2C,D). Each cluster contained identical gene order among three species as follows: ① *rrnL*-*rrnS*-*rps8*-*cob*-*rps3*-*nad4*, ② *rpl16*-*nad4L*-*nad2*-*cox2*-*atp4*-*atp6*, ③ *nad5*-*atp9*-*nad1*-*rps14*-*nad6* (Figure 2D). Four tandem gene clusters consisting of 2–4 genes were found to be common to three Isochrysidales species: (a) *rrnS*-*rps8*, (b) *rps3*-*nad4*, (c) *rpl116*-*nad4L*-*nad2*, (d) *atp9*-*nad1*-*rps14*-*nad6*. The relative arrangements of these collinear gene blocks revealed that at least one inversion and four translocations have occurred between *I. galbana* and *E. huxleyi*. One distinct difference between the two species was the insertion of a large complex tandem repeat region in *I. galbana*. Additional complete mitogenomes of new taxa in Isochrysidales order need to be sequenced and assembled for comparative genomic studies to confirm these conserved gene clusters and patterns in the arrangement of repeat regions. In contrast to the highly conserved or identical gene arrangement within orders in stramenopiles (Ševčíková et al., 2016; Liu et al., 2020; Sibbald et al., 2021), the haptophyte mitochondrial gene synteny analysis in the current study showed that multiple gene order rearrangements were detected within this lineage and even a given order, which is similar to cryptophytes (Kim et al., 2018).

### The *trans*-spliced gene *cox1*

**Figure 3:**
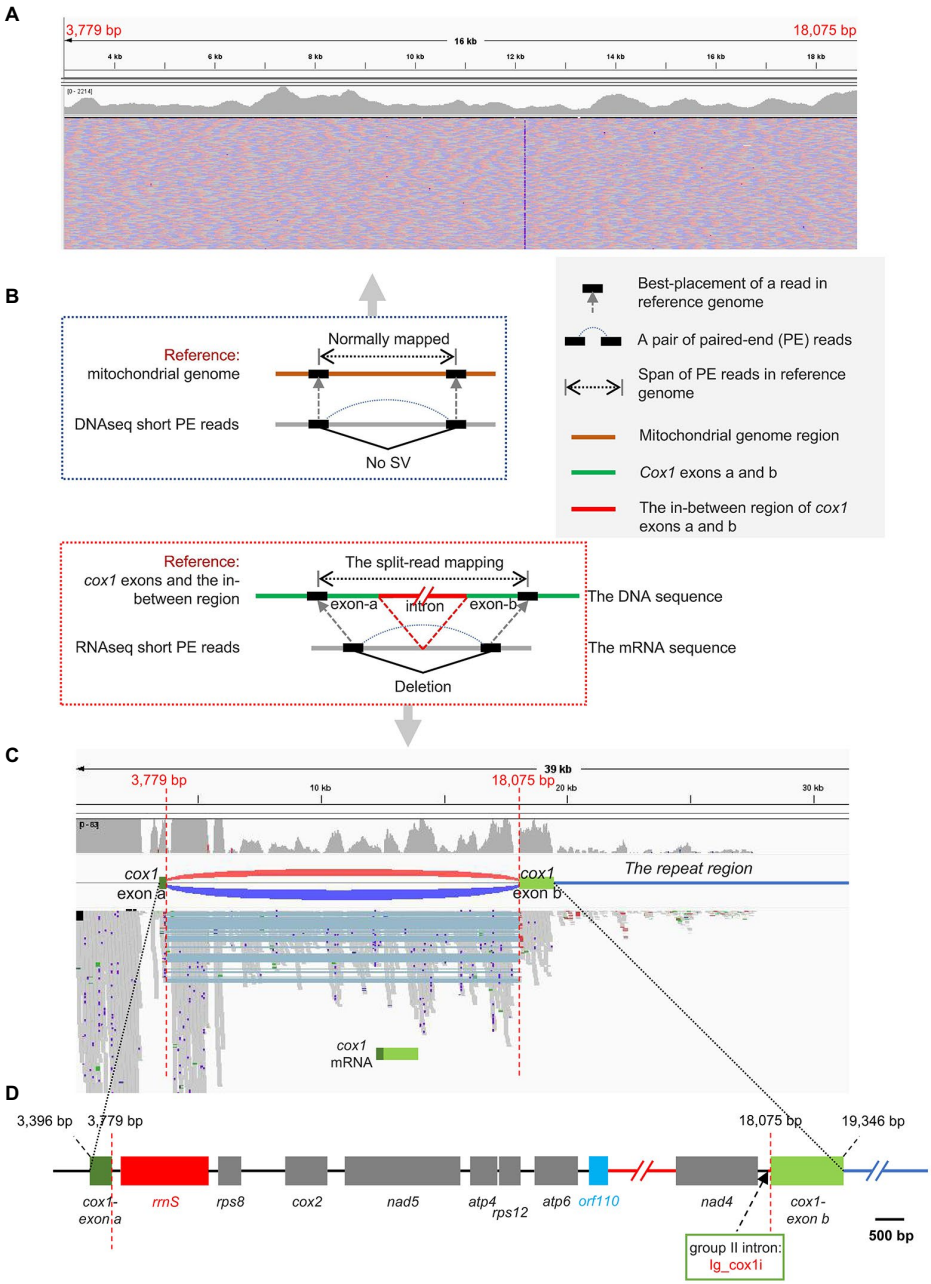
The split-mapping strategy to detect the two distantly dispersed exons in *cox1*. **(A)** Visualization of *I. galbana* mitochondrial genomic architectures by the IGV browser. DNA resequenceing paired-end reads were aligned to the mitochondrial reference genome. The paired-end mapping of high-throughput resequencing data revealed no obvious breakpoint was present at the borders of the exon a and b of *cox1*. **(B)** Principle of using high-throughput and massive paired-end mapping to detect distantly dispersed exons in *cox1*. The green lines represent two exons of *cox1* in the mitochondrial reference genome. The split-read mapping means reads span across a breakpoint of an SV. **(C)** Visualization of *I. galbana* mitochondrial transcriptome architectures by the IGV browser. RNA resequenceing paired-end reads were aligned to the mitochondrial reference genome. The discordant aligned read pairs from two *cox1* exons were visualized as red/dark-blue arcs, indicating the presence of deletion, while concordant paired-end reads were normally mapped with no arc in between. The split-mapping paired-end reads in the track contain the same deletion (light blue shades), which is consistent with the discordant pairs by arcs. Four exon borders of *cox1* in the reference sequence can be determined by accurate mapping of split reads. The *cox1* is the nearest neighboring gene to the repeat region (the dark-blue line). **(D)** Gene content and order in the region between two *cox1* exons in *I. galbana* mitochondrial genome. A novel gene *orf110* with unknown function was identified in the region between two cox1 exons at the position 12,111–12,443. A group II intron designated as Ig_cox1i was found adjacent to exon b of the *cox1* gene. The black and dark-blue line represent the intergenic region and the repeat region, respectively. The red line represents a region where a few genes (*cob-nad3-rps3*) were omitted.

The *cox1* gene encoding cytochrome *c* oxidase subunit 1 was the only one interrupted gene identified in this mitogenome. The *cox1* gene was split into two exons with 12 genes in between (Figure 2D). The Illumina RNA-seq data alignment result confirmed the presence of the two distantly dispersed exons of *cox1* in *I. galbana* (Figures 1, 3). Four exon borders of *cox1* could also be determined precisely by RNA-seq data (Figure 3C). The alignment of DNA-seq data generated from Illumina and PacBio platforms was also visualized and showed that no obvious breakpoint was present at the borders of the exon a and b of *cox1* (Figure 3A; Supplementary Figure S4), demonstrating the continuity and accuracy of the interrupted *cox1* gene and this assembly. Intriguingly, based on the location of *cox1* exons, we inferred the intron of *cox1* was removed by *trans*-splicing. The unusual structure of *cox1* in *I. galbana* is the first *trans*-splicing event ever observed in haptophyte mitochondrion. The mature *psaA* mRNA in the green alga *Chlamydomonas reinhardtii* chloroplast genome was also assembled by a process involving *trans*-splicing of three separate transcripts encoded at three widely scattered loci with many other protein-coding genes in-between (Goldschmidt-Clermont et al., 1991; Kück and Schmitt, 2021). Group II introns are believed to be critical for the splicing reaction and ubiquitously found in in the prokaryote genomes and the plant organelles which are derived from archaebacteria, but very rare or missing in green algal mitochondrion (Gray et al., 1998). Analysis of the whole mitogenome sequence identified one putative group II intron segment (located at the position 18,031–18,074) adjacent to exon b of *cox1*, which encoded no apparent open reading frame (designated as Ig_cox1i; Figure 3D). A novel gene *orf110* with unknown function was identified in the region between two *cox1* exons at the position 12,111–12,443. Group II introns have been identified in the mitochondrial genomes of various red algae, diatoms and haptophyte (Ehara et al., 2000; Nishimura et al., 2014; Guillory et al., 2018; Kim et al., 2018). A groupIIA intron with a classical intronic *orf* in the *cox1* gene was found in diatom species (Oudot-Le Secq and Green, 2011). How the group II intron involved in the process of *cox1 trans*-splicing and the mechanism behind the association remain to be solved. Further analysis should focus on identifying related genes/RNAs required in this process. Many nucleus-encoded pentatricopeptide repeat (PPR) proteins have been shown previously to be targeted to organelles and play essential roles in the *trans*-splicing of organelle introns (Lee et al., 2019; Kück and Schmitt, 2021). Shifts from *cis*- to *trans*-splicing show good correspondence with genome rearrangement rates (Guo et al., 2020), which is further evidenced by the highly poor gene cluster conservation among haptophyte mitogenomes (Figure 2A). On the contrary, no *trans*-splicing event has yet been detected in any mitochondria of the closely related taxa cryptophytes, whose gene order tend to be less variable (Kim et al., 2018). It should be noted that the *cox1* is the nearest neighboring gene to the repeat region (Figures 1, 3C). A hypothesis put forward here is that the intra-genomic rearrangement of *cox1* increased the instability of the mitogenome, enabling recombination at small repeats or nuclear DNA fragments to be readily integrated and accumulated into the double-strand break site. The NHEJ-DSB (Non-homologous end joining of double-strand breaks) repair mechanism was triggered when the DNA lesion such as DSB occurred (Waterman et al., 2020).

### RNA editing detection

Using a reference-based single-nucleotide polymorphisms (SNP) calling strategy, a total of 256,757 reads were aligned to the chloroplast genome and 8,660 reads aligned to the mitochondrial genome. Compared to the mitochondrial genome sequence, the aligned mitochondrial transcriptome differed by 6 SNPs (Supplementary Figure S5; Supplementary Table S10), among which two occurred outside of protein-coding regions and four were in exonic region of *atp6*, *rps3*, *nad6* and *rpl16*. Initial SNP calling results revealed these sites to be either real SNPs or caused by spurious deep sequencing. In higher plants, the nucleotide transition is commonly observed to be G/C to A/U (Steinhauser et al., 1999), whereas the range of nucleotides found in *I. galbana* mitogenome in the current study is extensive with more nucleotide transition types. Further strict screening found that each SNP has very low supportive depth with ref.:alt of 13:10, 9:4, 19:4, 37:4, 7:4, and 5:3, respectively (Supplementary Figure S5; Supplementary Table S10). We deduce that none of these SNPs were likely candidates for RNA editing. Likewise, no SNPs were identified when aligning RNA-seq data to *I. galbana* chloroplast genome sequence by SAMtools “mpileup” utility with the same parameters, revealing no chloroplast RNA editing event in *I. galbana*. Overall, the results based on deep transcriptome sequencing reflected that RNA editing does not occur in the organelles of *I. galbana*, which is in accord with the earlier observation in green algae (Cahoon et al., 2017) and Charales (Steinhauser et al., 1999) revealing the absence of RNA editing. The lack of RNA editing in *I. galbana* organelles is also consistent with the hypothesis that RNA editing has originated in embryophytes after they split from the ancestral algal lineage (Cahoon et al., 2017).

### Selective pressure analysis

The non-synonymous (*K*a) and synonymous (*K*s) substitutions and *K*a/*K*s ratio would reveal the natural selective strength for protein-coding sequence evolution (Yang and Nielsen, 2000). *Ka*/*Ks* value <1 is more prevalent given that synonymous nucleotide substitutions have occurred more frequently in protein-coding genes (Makalowski and Boguski, 1998). To pinpoint whether protein-coding genes within mitogenome underwent adaptive evolution in *I. galbana* compared with other Isochrysidales species. We compared the *Ka*/*Ks* ratio for 19 common protein-coding genes within mitogenomes between *I. galbana* and the most closely related species *E. huxleyi* hitherto found (Supplementary Tables S11, S12; Supplementary Figure S6). The *I. galbana* mitogenome was used as a reference.

The *K*a/*K*s ratios of protein-coding genes between two *E. huxleyi* mitogenomes were calculated to be zero or close to zero, which was in accord with synteny analysis results between two mitogenomes showing nearly no SNPs in these genes (Figure 2B). This result is common between two varieties of a single biological species. The average *K*a value and *K*a/*K*s ratio of 19 protein genes were fairly low (mean *K*a = 0.180 ± 0.12631; mean *K*a/*K*s = 0.042 ± 0.03888) between mitogenomes of *I. galbana* and *E. huxleyi* (Supplementary Table S12). The *K*a/*K*s ratios of all protein-coding genes between two species were less than one, providing the evidence that these genes were subjected to negative purifying selection among Isochrysidales species. More mitogenomes of Isochrysidales should be analyzed in the future to reach this conclusion.

Changes in evolutionary rates are strongly correlated with the gene function. All 19 genes exhibited rather low *K*a/*K*s values with 18 genes <0.08 (all <0.20). Out of them, seven genes revealed a rather low synonymous substitution rate (*K*a/*K*s < 0.02) between two species, which were found to mainly function in electron transport and ATP synthesis (Supplementary Table S11). The lowest *K*a/*K*s ratio was observed for three slow-evolving genes including the cytochrome b gene (*cob*), one cytochrome c oxidase gene (*cox1*) and one ATP synthase gene (*atp9*), suggesting they were conserved in Isochrysidales and play indispensable roles in haptophyte mitogenomes. Five genes exhibited highest *K*a/*K*s (> 0.06) values consisting of three genes encoding small subunits of ribosomal protein (*rps3*, *rps8*, *rps14*), one gene encoding NADH dehydrogenase subunit 2 (*nad2*) and one for ATPase subunit 4 (*apt4*), directly leading to a higher average *K*a/*K*s value for ribosomal protein genes (Supplementary Table S11). The *rps8* gene had the highest Ka/Ks ratio (0.180) in the mitogenome. Intriguingly, four out of these five genes (*atp4*, *rps3*, *rps8*, *rps14*) with slightly high Ka/Ks ratio were also absent from the core mitochondrial gene set (15 genes) identified in all haptophyte species aforementioned, suggesting rapid divergence has been occurred in these four genes in haptophyte mitogenomes in order to better adapt to environment.

### Gene content among algal mitochondrial genomes

**Figure 4:**
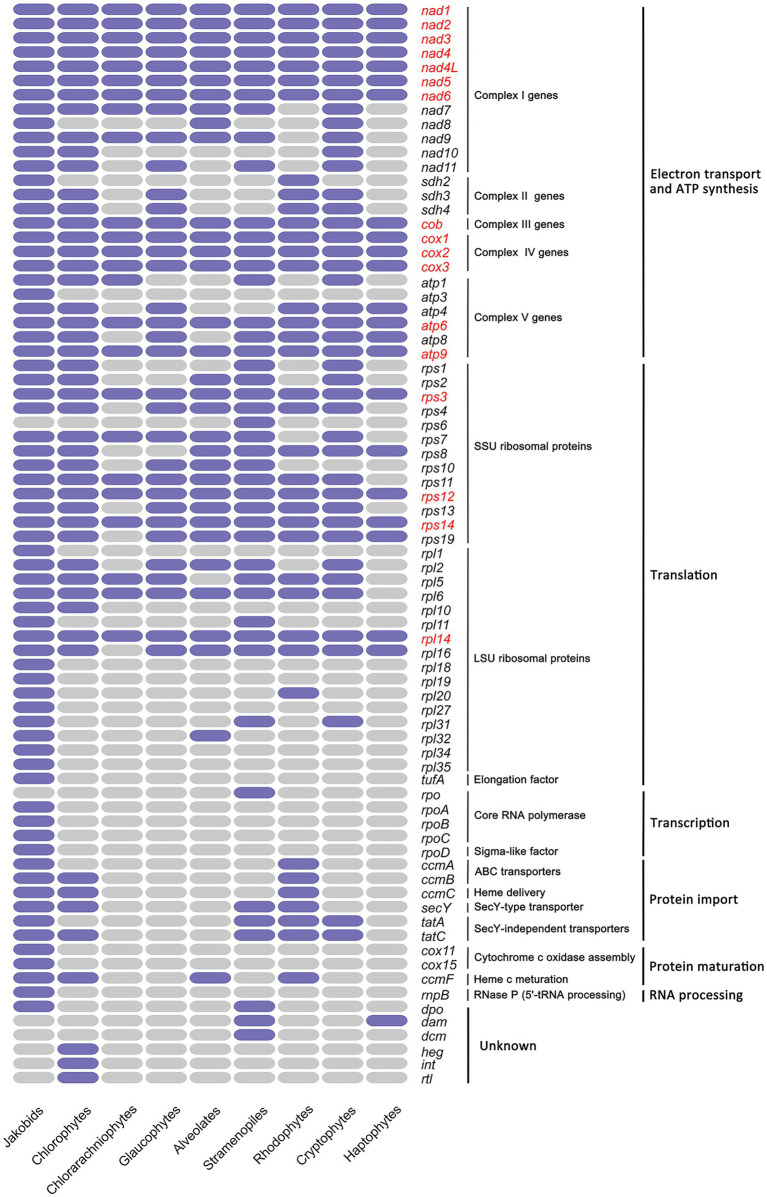
Heat map comparing gene content among the mitochondrial genomes of nine algal lineages. Only protein-coding genes with known functions are included. Purple color indicates the gene present in that group while grey color indicates absent; The 17 genes conserved among all groups are highlighted in red. Only one mitochondrial genome sequence (*Lotharella oceanica*) in Cercozoa (Chlorarachniophytes) that could be searched in GenBank. Complex I genes encode subunits of NADH dehydrogenase; Complex II genes encode subunits of succinate dehydrogenase; Complex III genes encode subunits of cytochrome bc1 complex; Complex IV genes encode subunits of cytochrome c oxidase subunits; Complex V genes encode subunits of ATP synthase.

Gene content of all mitogenomes from nine diverse eukaryotic assemblages are shown (Figure 4; Supplementary Table S13), including three primary algal lineages (Chlorophyta, Rhodophyta and Glaucophyta), four red alga-derived algal lineages (Cryptophyta, Alveolata, Stramenopiles and Haptista), one green alga-derived lineage Cercozoa (Chlorarachniophytes), and Jakobida which has exceptionally gene-rich mitogenomes. The mitogenomes of jakobid flagellates are noteworthy in retaining certain genes that were transferred to nucleus or absent in algal mitogenomes, including genes involved in cytochrome c oxidase assembly (*cox11*, *cox15*), genes encoding the ATP synthase subunit 3 (*atp3*), LSU ribosomal proteins (*rpl1*, *rpl18*, *rpl19*, *rpl27*, *rpl34*, rpl35), core RNA polymerase (*rpoA*, *B*, *C*, *D*), the RNA subunit of RNase P (*rnpB*) and elongation factor (*tufA*; Figure 4). With the exception of unknown ORFs in each mitogenome, the haptophyte mitogenomes contain the smallest conversed gene set (24 protein-coding genes) in algae, smaller than that observed in alveolates (33 protein-coding genes), glaucophytes (34 protein-coding genes), rhodophytes (38 protein-coding genes), cryptophytes (42 protein-coding genes), stramenopiles (45 protein-coding genes) and chlorophytes (48 protein-coding genes). Chlorarachniophytes mitogenomes were found to contain a small set of 24 protein-coding genes. This could be a consequence of only one mitogenome (*Lotharella oceanica*) that has been published to date. There were no records for euglenophytes and cyanophytes mitogenomes in NCBI. Despite core sets of genes of some groups have been noted before (Kim et al., 2018), their composition; however, present expanded in this study (glaucophytes [34 vs. 30], rhodophytes [38 vs. 22], chlorophytes [48 vs. 39] and haptophyte [24 vs. 22]) that could be ascribed to the addition of several newly updated mitogenomes in these groups since then. The core gene set of cryptophytes remain fairly constant despite the addition of several new mitogenomes. Excluding jakobids, a core set of 17 genes is present in mitogenomes of eight algal lineages sequenced to date.

Overall, a more comprehensive set of 48 genes was retained in green algae than that of red algae (45 genes), especially for genes involved in energy metabolism (NADH dehydrogenase subunits) and translation (ribosomal proteins; Figure 4). In contrast, mitogenomes in the red lineage retained genes encoding the translocase subunit *tatA* related to twin-arginine translocation system transporters, the succinate dehydrogenase subunit (*sdh2*), the cytochrome *c*1 ABC transporter ATP-binding subunit (*ccmA*) and the ribosomal protein L20 (*rpl20*). The smallest gene set of haptophytes mitogenomes suggest that they have undergone an extreme mitogenome reduction compared to others. Despite of the minimal set, the majority of the haptophytes mitochondrial genes were implicated in oxidative phosphorylation (“OXPHOS”) and protein synthesis belonging to five mitochondrial respiratory complexes, as described above.

Jakobid and cryptophytes mitogenomes have the largest complement of genes encoding NADH dehydrogenase subunits (*nad1*–*nad11*), which are combined into the complex I in mitochondrion (Figure 4). By contrast, the *nad7*–*11* genes were missing from all rhodophytes mitogenomes and typically rarely found in many species of other phyla. Three genes, *nad7*, *nad9*, *nad11*, were present in stramenopiles, glaucophytes and chlorophytes. Chlorophytes have an additional *nad10* compared with the other two. Instead, alveolates mitogenomes have *nad8* superseding *nad11* in this regard.

Mitochondrial respiratory complex II are composed of four subunits of the succinate dehydrogenase, two of which, encoded by *sdh1* and *sdh2* genes, are hydrophilic and form a subcomplex to play a catalytic role; the other two (encoded by *sdh3* and *sdh4*) are hydrophobic and membrane-integral subunits, playing specific functions in electron transfer (Huang et al., 2019). Previous studies have found that *sdh1* and *sdh2* are highly conserved among species and has already transferred to the nuclear genome in almost all eukaryotes (Lang et al., 1999; Huang et al., 2019). According to our result, *sdh1* was absent in the mitogenomes of any algal lineage we examined, suggesting that this gene might experience an ancient transfer event. Although *sdh2* is nucleus encoded in almost all algal species, it is found to be mtDNA-encoded in Rhodophyta and Jakobida, which is in accordance with previous findings (Gray et al., 1998; Burger et al., 2013; Huang et al., 2019). The other two complex II subunits *sdh3* and *sdh4*, on the contrary, were found in either nuclear encoded or mitochondrion encoded (Gray et al., 1998), and our result also corresponds well with this trend. It should be noted that all haptophytes, stramenopiles and alveolates have lost all *sdh* genes from their mitogenomes. This is similar to the observation in animals and fungi that present all four *sdh* genes have moved to the nuclear genome (Huang et al., 2019).

Genes encoding the cytochrome *bc1* complex subunit (complex III: *cob* gene) and three cytochrome *c* oxidase subunits (complex IV: *cox1*, *cox2* and *cox3* genes) were present in the mitogenomes of all haptophyte species and other examined algal lineages. These four genes were dispersedly distributed throughout the entire mitogenome in Haptophytes (Figure 2), in contrast to the observation in cryptophyte mitogenomes, which showed that *cob* and *cox* genes normally stayed in groups (Kim et al., 2018). Group II introns were particularly common in the mitochondrial *cox1* gene of various red algae, stramenopiles, cryptophytes (Guillory et al., 2018; Kim et al., 2018; Sibbald et al., 2021). Like *Chroomonas placoidea* in cryptophytes, the *cox1* gene in *I. galbana* was split into two exons with many genes in-between (Kim et al., 2018). In *I. galbana*, a putative group II intron segment with no apparent ORF was also found adjacent to the exon b of *cox1*, and one single ORF with unknown function lay in the middle region between two cox1 exons (Figure 3D). This has not been consistently observed in other algal species which reported that many mitochondrial group II introns of *cox*1, *cob* or *rnl* genes often harbor protein-coding regions corresponding to intron encoded proteins (IEPs), which contain domains encoding a reverse transcriptase/maturase, DNA binding and DNA endonuclease (Zimmerly et al., 2001; Kim et al., 2018; Sibbald et al., 2021).

The biggest complement of ATP synthase genes (complex V genes) was possessed by jakobid mitogenomes (six *atp* genes). The *atp3* gene was absent in all eight algal lineages. In algal groups, all green algae and cryptophytes possessed most of the *atp* gene set (five *atp* genes) while the alveolates contained the minimal *atp* gene set (*atp6* and *apt9*). Excluding alveolates and chlorarachniophytes, three *atp* genes (*atp6*, *atp8*, *atp9*) were conserved in the mitogenomes of other algal lineages.

The twin-arginine protein transport pathway (Tat pathway) are ubiquitously present in prokaryotes and plant organelles, and two *tat* genes (*tat*A*, tat*C) were found to be generally conserved in diverse prokaryotes, plastids and some mitochondria (Berks, 2015; Palmer and Stansfeld, 2020). The current study noted their presence in the mitogenomes of three red-algal lineages (rhodophytes, cryptophytes, stramenopiles) aside from jakobids (Figure 4). In the green-algal lineages, the mitochondrial *tat*C homologs were present but no *tat*A was found. It is worth noting that both *tat* genes were absent from two red-algal lineages (haptophyte, alveolates) and glarucophytes mitogenomes sequenced to date (Figure 4). Based on our previous finding, both *tat* genes were also absent in three haptophyte chloroplast genomes sequenced to date (Puerta et al., 2005; Fang et al., 2020). The general secretory signaling (Sec) pathway always operate in parallel with the Tat pathway to transport folded proteins across membranes (Palmer and Stansfeld, 2020). The *secY* gene involved in Sec pathway was limited to chloroplast genomes in haptophytes (Fang et al., 2020) while it still existed in green-algal, red-algal and stramenopiles mitogenomes (Figure 4). Other four genes (*tuf*A and *rpo*A/B/C) that have been absent from all algal mitogenomes (Figure 4) were found in chloroplast genomes of haptophyte species (Fang et al., 2020). Clearly more mitogenome data are required from mitogenome sequences lacking lineages such as haptophytes and chlorarachniophytes to confirm the trend.

### Phylogeny inference

**Figure 5:**
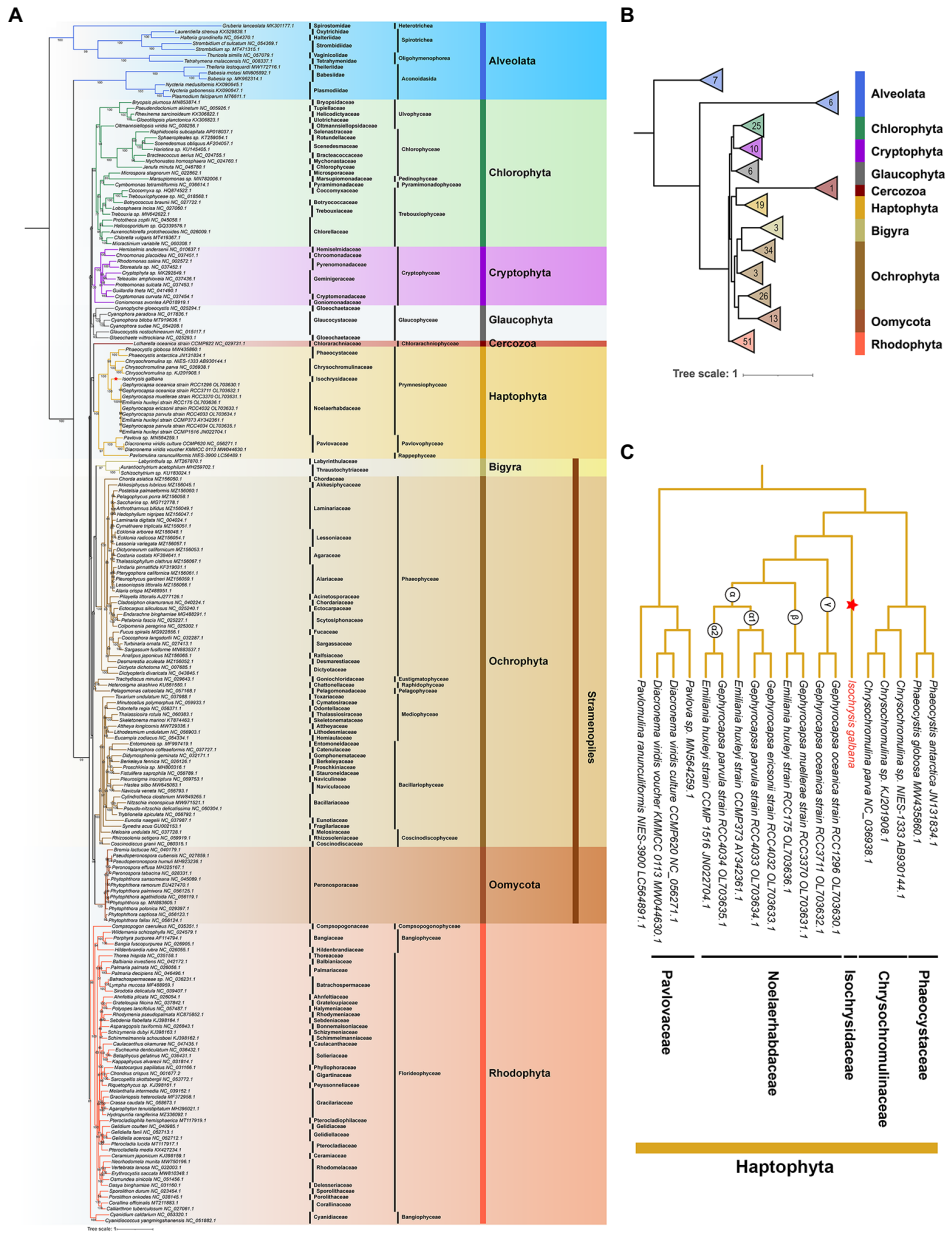
Maximum-likelihood (ML) phylogenetic analysis of COX1 proteins. **(A)** The ML phylogenetic tree constructed by COX1 coding sequences of 204 mitogenomes. The ML phylogenetic tree was constructed with coding sequences of the single-copy gene *cox1* from 204 selected mitogenomes of eight lineages (10 algae phyla) consisting of five red-algal lineages (Cryptophyta, Alveolata, Stramenopiles, Haptophyta and Rhodophyta), two green-algal lineages (Chlorophyta and Cercozoa) and Glaucophyta. Taxa are colored according to the corresponding phylum. Maximum-likelihood bootstrap support are indicated at nodes. NCBI GenBank accession numbers were listed next to their corresponding species. Scale bar represents amino acid substitutions per site. **(B)** Main phylogenetic topology of 10 algae phyla inferred by COX1 proteins. **(C)** Phylogenetic relationships of 18 haptophyte mitogenomes based on COX1 proteins.

The mitochondrial maximum-likelihood (ML) phylogenetic tree was constructed using a total of 204 non-redundant COX1 amino acid sequences recovered from GenBank and the *I. galbana* mitogenome (Figure 5; Supplementary Table S14). The multiple-gene ML tree was also inferred with concatenated amino acid sequences of 10 common single-copy protein-coding genes from 183 mitogenomes (Supplementary Figures S7, S8). Our extensive taxon sampling suggested that all lineages present well-resolved internal relationships. The topology within the haptophyte clade was very similar to the phylogenetic reconstructions inferred from individual genes or entire plastome/mitogenome sequences (Figures 2A, 5; Supplementary Figure S7; Bendif et al., 2013; Fang et al., 2020; Kawachi et al., 2021; Song et al., 2021; Kao et al., 2022). Within the monophyletic clade of Haptophyta, two main clades consisting of three classes (Prymnesiophyceae, Pavlovophyceae and Rappenphyceae) were statistically strongly supported (MLBS ≥ 97%; Figure 5). *Isochrysis galbana*, *E. huxleyi* and four *Gephyrocapsa* speices (*G. oceanica*, *G. muellerae*, *G. parvula*, *G. ericsonii*) were nested within the Isochrysidales monophyletic clade, while *I. galbana* was the only species found in the family of Isochrysidaceae (Figure 5C). This is consistent with previous phylogenetic analyses on Isochrysidales inferred from a concatenated sequence of three genes (SSU/LSU rDNA and *cox1*) and the entire plastome coding sequences (Bendif et al., 2013; Fang et al., 2020). The interlaced relationship of *Emiliania-Gephyrocapsa* mitogenomes (Figure 5C) was largely congruent with the topology inferred by concatenated orthologous coding genes of entire mitogenomes (Kao et al., 2022), which also revealed that the most divergent lineage (γ) harbored the mitogenomes of *G. oceanica* RCC3711 and RCC1296, and *G. muellerae* RCC3370 and *E. huxleyi* RCC175 were nested within the β lineage, while the α lineage (subdivided into α1 and α2) contained the rest species. The inclusion of the novel species *P. ranunculiformis* NIES-3900 of the new class Rappenphyceae within Haptophyta was strongly supported (MLBS = 97%), which was the first to diverge from Prymnesiophyceae and formed a sister group to the class of Pavlovophyceae. This is slightly inconsistent with the ML tree inferred from the mitochondrial dataset that was composed of 49 taxa, which revealed the sister relationship between NIES-3900 and Prymnesiophyceae (Kawachi et al., 2021). The much larger dataset we used in our phylogenetic analysis could explain the discrepancy. The COX1 identity of alveolates was found to the exclusion of all other four red-derived algal lineages (cryptophytes, stramenopiles, haptophytes and rhodophytes; Figure 5A). Alveolates COX1 formed two distinct branches before the split of rhodophytes and other lineages with secondary red-derived plastids. The positions of alveolates and stramenopiles contradicted the formation of SAR supergroup (Keeling and Burki, 2019) or the finding that stramenopiles diverged from alveolates and their extant plastids are direct descendants of a common red algal plastid (Janouškovec et al., 2010), suggesting a different evolutionary trajectory of *cox1* genes. This could be explained by mitochondrial-plastid or single/multiple genes phylogenomic incongruence. A monophyletic stramenopiles clade was formed by three distinct phyla (Bigyra, Ochrophyta, and Oomycota), which with nearly full support branches as sister to Haptophyta (Figure 5; Supplementary Figures S7, S8). This relationship is in accord with the schematic tree based on a consensus of phylogenomic studies together with morphological characteristics (Keeling and Burki, 2019). The Cercozoa (chlorarachniophytes) was unexpectedly grouped into the clade of Haptophyta (Figure 5) or the clade of stramenopiles (Supplementary Figures S7, S8; both were the red-derived lineages), in contrast to the perception of its green algae-derived origin. The similar trend was also found in recent algal phylogenetic analysis based on conserved BUSCO sequences (Shi et al., 2021). The cryptophyte lineage was located separately from the SH lineages (Figure 5; Supplementary Figures S7, S8), which is strongly in line with earlier phylogenetic analysis based on a dataset of 16 conserved mtDNA proteins (Kim et al., 2018). In particular, at odds with the evolutionary hypothesis supported by morphological, molecular and organelle phylogenetic studies that the single event of primitive primary endosymbiosis led to the divergence of archaeplastids taxa (green algae, red algae and glaucophytes), the red-derived lineage cryptophytes with secondary plastids formed a monophyletic clade with lineages with primary plastids (Figure 5; Supplementary Figures S7, S8). This finding is also in line with many previous findings that the archaeplastids group are mostly interrupted by cryptists (Keeling and Burki, 2019). To this day the monophyly of archaeplastids group still remain controversial due to lack of comprehensive and solid support from most molecular trees. Compared with the COX1 tree, the main phylogenetic topology of nine algae phyla of the multiple-gene tree was more consistent with the schematic eukaryotic tree summarized by many phylogenomic studies (Keeling and Burki, 2019). From mitochondrial perspective, the relative relationship among algal lineages implied their considerably deeper divergence rather than a simple origin, which fit with the “multiple eukaryote-eukaryote endosymbiosis (EEE) hypothesis” or “rhodoplex hypothesis” hypothesis (Archibald, 2009).

## Conclusion

In this study, we reported the first full-length mitogenome of *I. galbana*, which is an ~39,258 bp circular molecule with an AT-rich pattern (72.9%), encoding 20 protein-coding genes, 24 unique tRNA genes and two rRNA genes. This mitogenome consists of an elaborate combination of direct repeats (about 12.7 kb) uninterrupted by genes, making it much larger than most other haptophytes mitogenomes. Comparative analysis of haptophyte mitogenomes revealed that they shared an identical complement of 15 energy and metabolism genes, exhibited opposite or same strand polarities within different orders and had poorly conserved gene content and order. Genes were broadly conserved with the same strand orientation but gene order was highly variable in the Isochrysidales order. The *Ka/Ks* ratios of all common genes in Isochrysidales mitogenomes were less than one, suggesting that they are under purifying selection. The visualization of RNA-seq and DNA-seq alignment reads verified the present of the *trans*-spliced gene *cox1* that contained two distantly dispersed exons in *I. galbana*. This is the first *trans*-splicing event ever identified in mitochondrion of haptophytes. The high mitogenome rearrangement rates in haptophytes could account for the shifts from *cis*- to *trans*-splicing. Also, the intragenomic rearrangement of *cox1* could increase the genome instability, thus accelerate the multimerization and accumulation of pre-existing small-repeats at the site of DNA damage. No organelle RNA editing was found in *I. galbana* based on deep transcriptome sequencing data, further confirming the perception that RNA editing evolved after embryophytes separated from the algal ancestry of all land plants. Mitogenome comparison among algal lineages revealed haptophytes contained the most contracted protein-coding gene set. Haptophytes mitogenomes have lost many functional genes (e.g., *sdh*, *tat*, and secY genes) in comparison with other red-lineages. The distinct phylogenetic relationship reflected by chloroplast and mitogenome genes underscore their dramatic different evolutionary tempo and pattern even they coexist in the same cell.

## Data availability statement

The final complete mitogenome sequence with gene annotation has been deposited in the NCBI GenBank under accession number of ON688523. The Illumina DNA resequencing raw reads in FASTQ format of I. galbana genome have been deposited in the Genome Sequence Archive database (GSA; https://ngdc.cncb.ac.cn/gsa/) under accession number of CRA007102. The Illumina RNA-sequencing raw data of I. galbana transcriptome under different conditions are available in GSA under accession number of CRA007103.

## Author contributions

JF conceived this mitogenome project and coordinated the research activities. JF and XX designed the experiments and wrote the manuscript. JF and AL assembled and annotated the mitogenome. QC and JF carried out the experiments and processed genome and transcriptome resequencing data. JF, XX, AL, QC, and SL performed the bioinformatic analyses. All authors contributed to the article and approved the submitted version.

## Funding

This work was supported by the Natural Science Foundation of China (grant number 41906096), the Middle-aged Teachers from Fujian Provincial Department of Education (grant number JT180082), the Natural Science Foundation of Fujian Province, China (grant number 2019J05066) and Key Projects of Science and Technology Bureau of Fuzhou, Fujian, China (grant number 2021-N-119).

## Conflict of interest

The authors declare that the research was conducted in the absence of any commercial or financial relationships that could be construed as a potential conflict of interest.

## Publisher’s note

All claims expressed in this article are solely those of the authors and do not necessarily represent those of their affiliated organizations, or those of the publisher, the editors and the reviewers. Any product that may be evaluated in this article, or claim that may be made by its manufacturer, is not guaranteed or endorsed by the publisher.

## Footnotes

^1^ http://zombie.cb.k.u-tokyo.ac.jp/sprai/

^2^ https://www.snapgene.com/

^3^ http://www.bioinformatics.nl/cgi-bin/emboss/cusp
