# Supplementary material for: The complete mitochondrial genome of Isochrysis galbana harbors a unique repeat structure and a specific trans-spliced cox1 gene
Source: Front Microbiol. 2022 Sep 27;13:966219. doi: 10.3389/fmicb.2022.966219 (PMC9551565; doi:10.3389/fmicb.2022.966219)
Supplement: Supplementary file 15 [file Data_Sheet_1.DOCX]

Supplementary Material

## Supplementary Figures

**
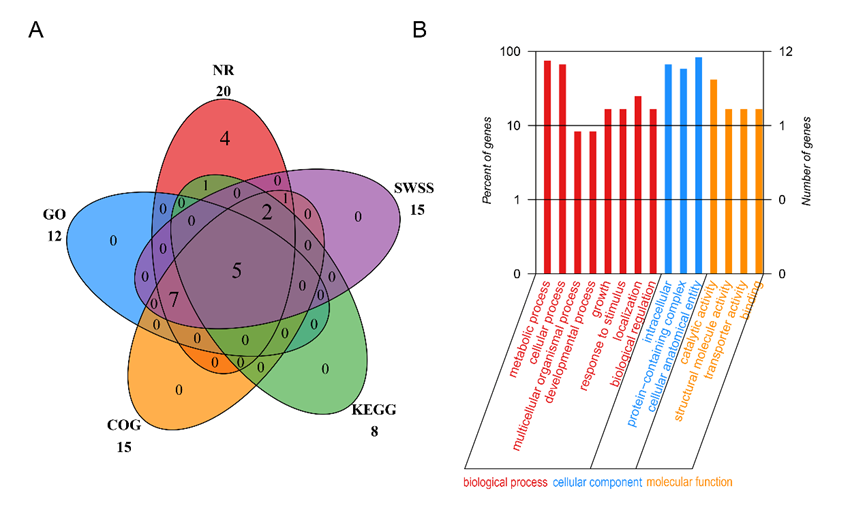
**

**Figure S1 Functional annotation of *I. galbana* mitochondrial protein coding genes.** **A.** Functional annotations in five integrated protein sequence databases. The protein-coding genes was carried out in following five integrated protein sequence databases: Nr (NCBI non-redundant protein database), Swiss-Prot, COGs (Clusters of Orthologous Groups), KEGG (Kyoto Encyclopedia of Genes and Genomes) and GO (Gene Ontology) databases. **B.** Level 2 GO annotation of 20 protein-coding genes. The assigned GO terms belonged to three major ontologies: molecular function (MF), biological process (BP) and cellular component (CC). The pie-charts show the number of genes that were assigned to the corresponding functional categories.

**
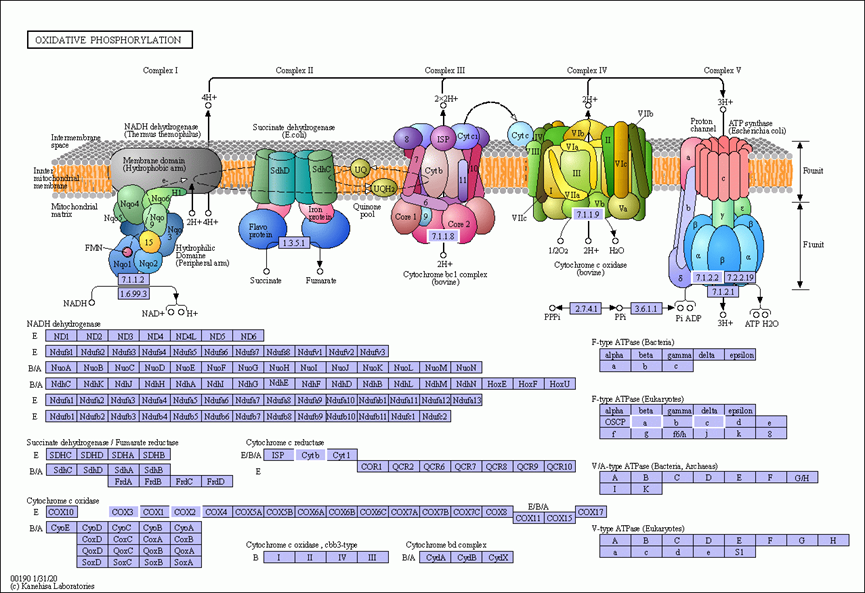
**

**Figure S2 Oxidative phosphorylation (ko00190) pathview of** ***I. galbana* mitochondrial protein coding genes.** Five genes (*Cytb*, *cox2*, *cox3*, *atpa*, *atpc*) represented by white boxes indicate genes associated with oxidative phosphorylation pathway.


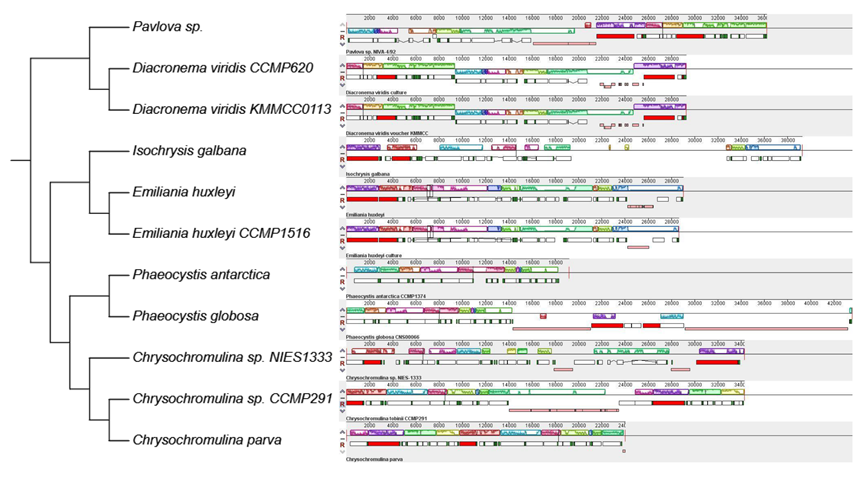


**Figure S3 Gene map comparison of eleven haptophyte** **mitochondrial genomes based on Mauve alignment.** Inside each block a sequence identity similarity profile is shown. Individual genes and strandedness are shown below each genome block. Red regions in the individual gene plots indicate the locations of the ribosomal operon comprising the 16S and 23S rRNA genes (rrnS and rrnL).


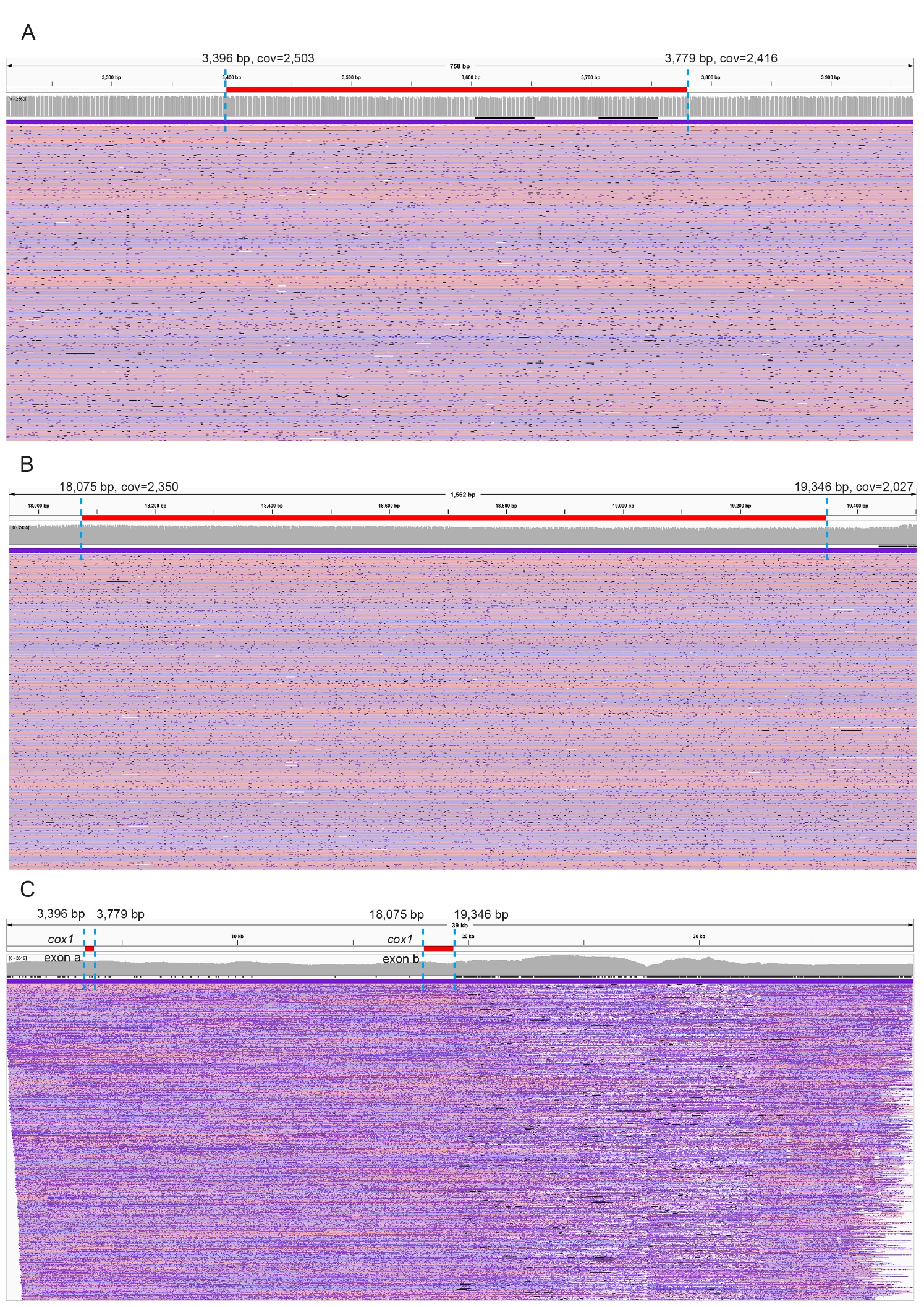


**Figure S4 Visualization of** ***I. galbana* mitochondrial genomic architectures based on the alignment of PacBio subreads by the IGV browser.** PacBio long subreads were aligned against the *I. galbana* complete mitochondrial genome using minimap2 (Li, 2018). Visualization of the PacBio subread mapping with high resolution among the four exon borders showed that no obvious breakpoint was present at the four borders of the exon a and b of *cox1*. The mapping depths for these four borders were 2,503x, 2,416x, 2,350x and 2,027x respectively. The assembly continuity and accuracy of regions harboring *cox1* was confirmed by the alignment of PacBio subreads.


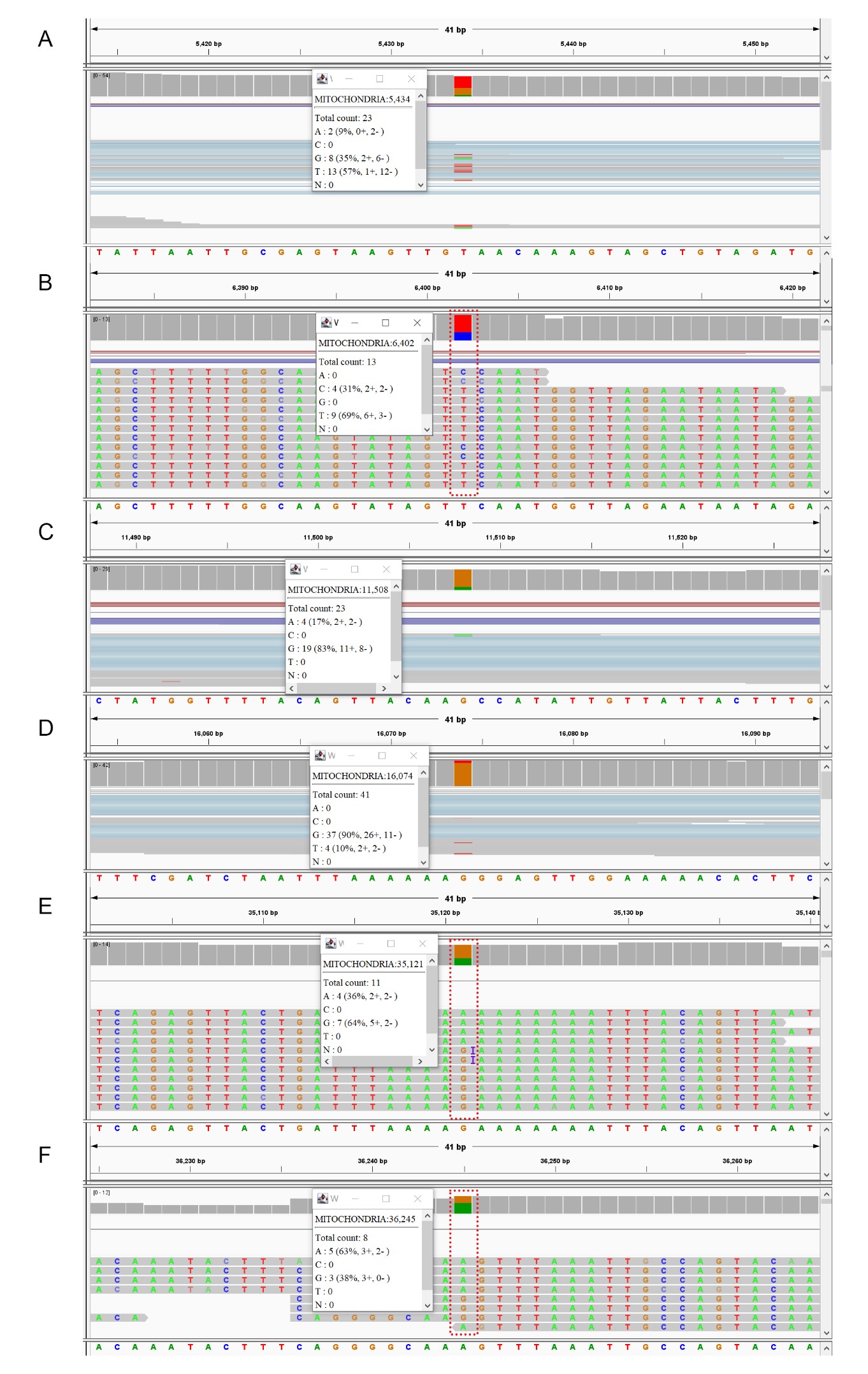


**Figure S5 Alignment of deep sequenced transcriptome reads to the *Isochrysis galbana* mitochondrial genome revealing six putative SNPs (A-F).** The mRNA was isolated from *I. galbana* 3011 which was the same variety used for genome sequencing. After mapping and filtering, a total of 8,660 reads were aligned to the mitochondria genome. The six putative edit sites were detected at bases 5,434, 6,402, 11,508, 16,074, 35,121, 36,245 and and manually visualized in the IGV software. The SNP sites with read depth ≤20 were viewed by all bases. The exact number of reads supporting reference or alternate alleles were shown in pop-up boxes.


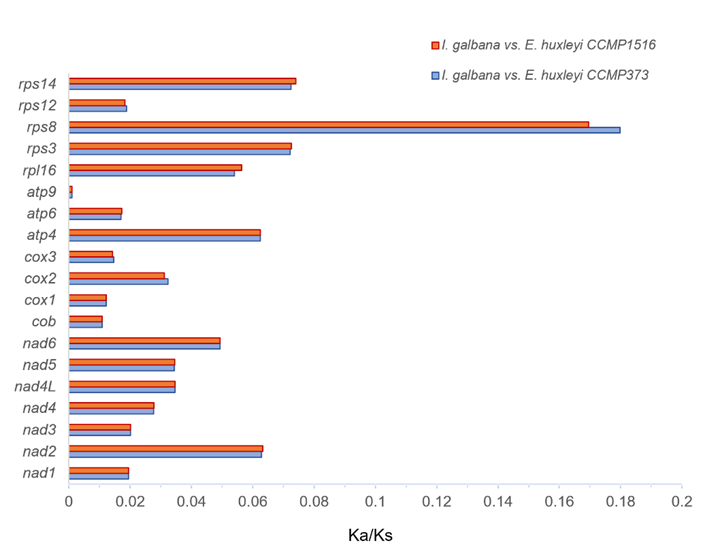


**Figure S6 Ka/Ks ratios of nineteen protein-coding genes in two *E. huxleyi* species compared with *I. galbana*.** Red boxes indicate the Ka/Ks ratios for *I. galbana* vs. *E. huxleyi CCMP1516*; Blue, *I. galbana* vs. *E. huxleyi CCMP373*.

**
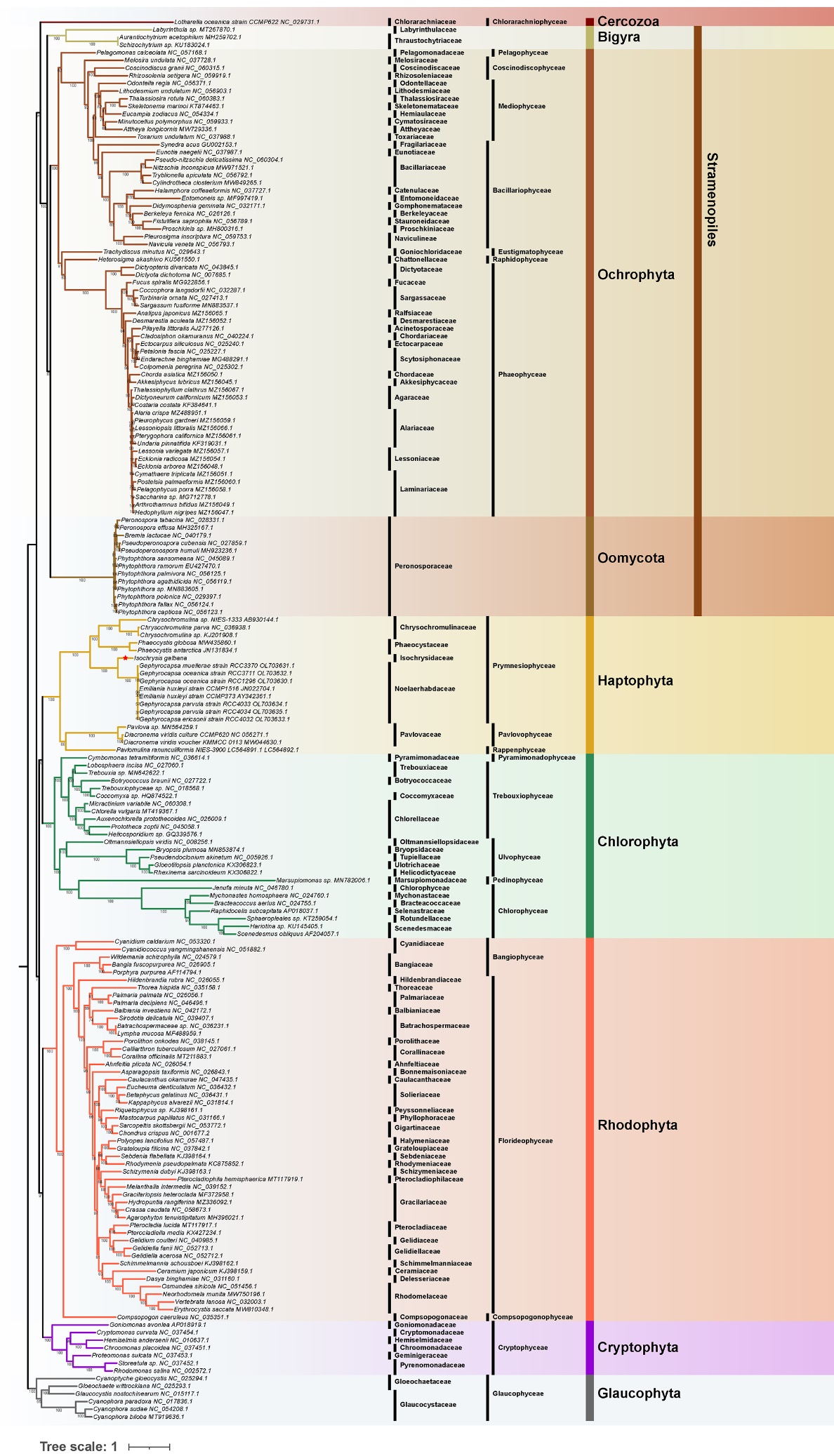
**

**Figure S7 Maximum-likelihood (ML) phylogenetic analysis of** **multiple-gene protein sequences of 183 mitogenomes.** The concatenated mitochondrial dataset comprised ten common single-protein (nad1, nad2, nad3, nad4, nad4L, nad5, nad6, cob, cox1, atp6) among 178 species (183 mitogenomes) and 6,266 amino acid positions in total. The mitogenomes of 178 species in green-algal lineage Chlorophyta (24 species) and Cercozoa (1 species), Glaucophyta (6 species), and five red-algal lineages consisting of Cryptophyta (7 species), Stramenopiles (77 species) and Haptophyta (13 species) and Rhodophyta (50 species) were retrieved from NCBI GenBank. Scale bar represents amino acid substitutions per site.

**
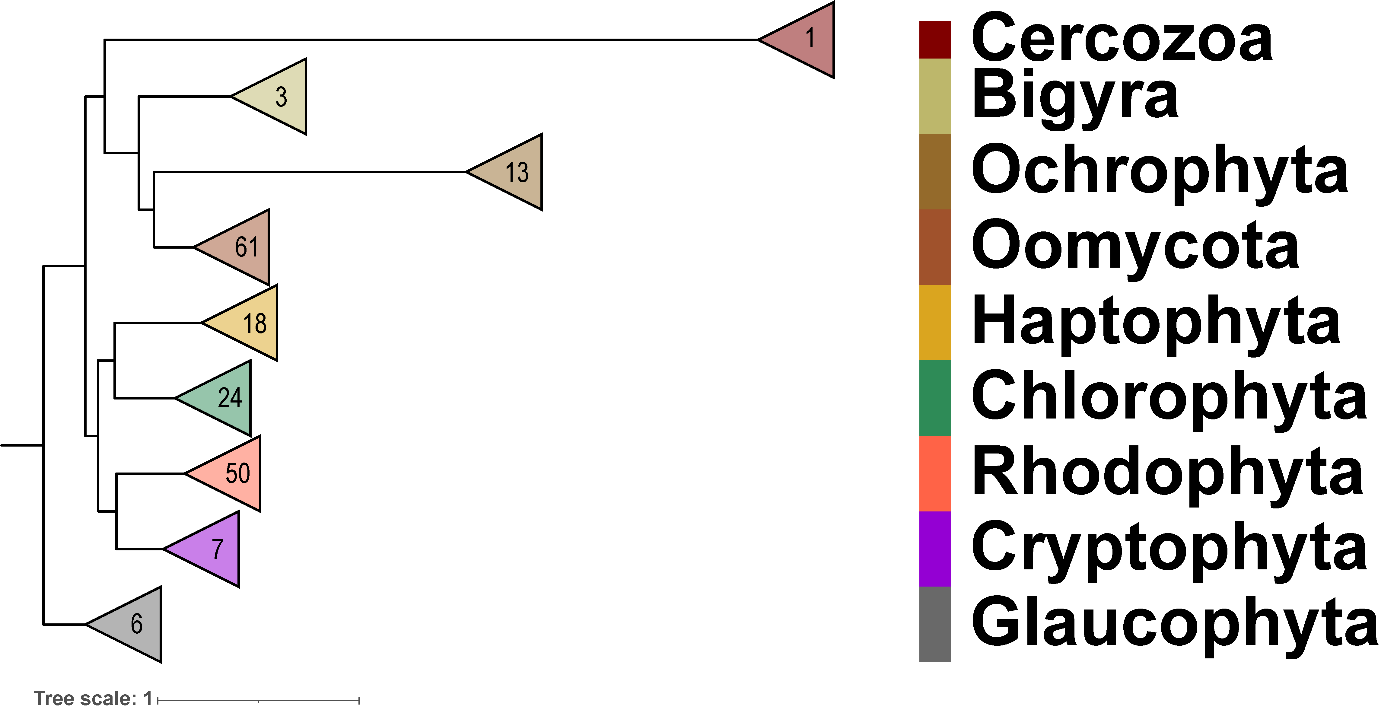
**

**Figure S8 Main phylogenetic topology of nine algae phyla inferred by multiple-genes proteins** **sequences of 183 mitogenomes.**
